# The Risk Genes S1PR5, CMC1, and ASAH1 as Potential Targets for the Diagnosis, Immunotherapy, and Treatment of Colon Adenocarcinoma by Single-Cell and Bulk RNA Sequencing Analysis

**DOI:** 10.2174/0109298673331144241009082052

**Published:** 2024-11-07

**Authors:** Zipeng Xu, Jiantao Gong, Weidong Hu, Chen Ge, Genxi Tong, Fengjun Cai, Zhenghai Zhu, Yihang Yuan, Chaobo Chen

**Affiliations:** 1 Department of General Surgery, Xishan People’s Hospital of Wuxi City, Wuxi, 214105, China;; 2 Department of Gastroenterology, Xishan People’s Hospital of Wuxi City, Wuxi, 214105, China;; 3 Department of General Surgery, Affiliated Hospital of Medical School, Nanjing Drum Tower Hospital, Nanjing University, Nanjing, 210008, China

**Keywords:** Colon adenocarcinoma, scRNA-seq, bulk RNA-seq, ICF model, immunotherapy, therapeutic targets, S1PR5, CMC1, ASAH1

## Abstract

**Objective:**

Globally, one of the main causes of cancer-related mortality is Colon Adenocarcinoma (COAD). In this study, a new special Immune Cell Functions (ICF) risk model was constructed using single-cell and bulk RNA sequencing data to develop a new understanding and clinical applications for COAD.

**Methods:**

The immune function gene sets were downloaded from a literature reference, and the COAD single-cell dataset GSE146771 was downloaded from the Tumour Immune Single Cell Hub database. Using Lasso analysis, a multiple gene signature was made from the enrichment scores of immune function gene sets that were enriched in different ways. Robust validation of the signature was then performed in multiple independent cohorts. After that, we built the model using a 10-fold cross-test and evaluated its independence for clinical usage using a nomogram. We also investigated the connection between signature and immune function, genetic variation, immunotherapy, and the cancer immunological microenvironment. Lastly, we used qPCR and immunohistochemistry to examine the expression of the unreported model genes. To find the regulatory functions of unreported model genes, an EdU assay was employed.

**Results:**

First, 20 differentially enriched immune function gene sets were identified. Ten genes can be used as a risk profile to assess the prognosis of colon cancer, according to Lasso regression analysis. Signature performance was stable in both the training cohort and two independent GEO external cohorts, and risk scores were confirmed as independent prognostic factors. At the same time, our risk model continued to be highly predictive across various clinical clusters and clinical characteristics, such as immune checkpoints, tumour genome mutations, and chemotherapeutic drug resistance. Patients in the low-risk group have exhibited a higher chance of benefiting from immunotherapy, according to immunotherapy response research. qPCR and immunohistochemistry analysis have revealed S1PR5 expression as high in COAD tissues, while CMC1 and ASAH1 expression has been found to be low. According to the findings of the functional experiment, S1PR5, CMC1, and ASAH1 may control the ability of CRC cells to proliferate.

**Conclusion:**

In this study, using scRNA-seq and bulk RNA-seq data, we created a risk model to predict the prognosis and effectiveness of immunotherapy in patients with COAD. In addition, we have discovered three model genes (S1PR5, CMC1, and ASAH1) that have not been reported before. These genes have the potential to be novel therapeutic targets in Colorectal Cancer (CRC). These findings suggest that this model could be used to evaluate the prognostic risk and identify potential targets for COAD patient treatment.

## 1 INTRODUCTION

Colorectal Cancer (CRC) has risen to second place in China’s overall incidence rate, ranking fifth among the leading causes of cancer death. The incidence and mortality rates have increased significantly compared to the past, and the health threat has become more serious [1, 2]. When CRC is diagnosed, about 20% of patients already have metastatic disease. Furthermore, up to 50% of patients with localised diseases eventually develop metastasis [3, 4].

Recent advances in the treatment of Colorectal Cancer (CRC) have significantly improved patient outcomes through the development of targeted therapies, immunotherapy, and precision medicine [5, 6]. Biomarker-based stratification for personalised therapy is a big step forward. This is especially true for metastatic CRC, where targeted treatments, like encorafenib combined with cetuximab, have been shown to work in patients with BRAF mutations [7]. Immune checkpoint inhibitors, like nivolumab, ipilimumab, and pembrolizumab, have also been shown to help treat MSI-H and Lynch syndrome-associated CRC. Improving chemotherapy treatments, like FOLFOX and FOLFIRI, along with new technologies, like liquid biopsies, for early detection and monitoring, has made it easier for people with CRC to survive and get better care [8, 9].

The prognosis is dire for the majority of patients, even though these thorough and precise treatment plans increase the overall survival duration of advanced diseases to three years [10, 11]. Patients with colorectal cancer face challenging circumstances and a persistently poor long-term survival rate. It is critical to investigate the mechanism of COAD cell invasion and metastasis [12]. Therefore, scientists have been working to reveal the complex molecular mechanism of COAD occurrence, development, and invasion to provide opportunities to improve clinical outcomes and treatment plans.

An increasing amount of research has focused on the expression of genes connected to tumours that alter during processes, such as tumour occurrence, development, invasion, and metastasis. This is due to the improvement of various high-throughput sequencing methods and the creation of public biological information databases. Second-generation sequencing technology is used to select tumour markers [13]. The complex ecology that includes stromal cells, cancerous cells, and other cell types is known as the Tumour Microenvironment (TME) [14]. It is highly heterogeneous and significantly impacts tumour growth, metabolism, metastasis, and even the therapeutic effect of anti-cancer drugs [15, 16]. New studies have shown that immune genes can be used to help figure out who is at risk for breast, stomach, thyroid, and ovarian cancer and what will happen to them [17-21]. As a result, TME significantly influences the genetic characteristics of immunotherapy effectiveness and patient prognosis. However, COAD's mechanism and prognostic value remain unknown. Therefore, more research is needed, which may aid in developing new and more effective treatment strategies.

In cell sequencing, bulk RNA-seq is a commonly employed technique. It extracts and breaks up DNA from every cell in the tissue [22]. The data from bulk RNA-seq represent the average gene expression in each tissue's cells. Compared to bulk RNA-seq, single-cell RNA sequencing (scRNA-seq) is a potent technique for analysing the intricacy of solid tumors [23]. It makes it easier to study how different cells work and gives detailed descriptions of cell variety and heterogeneous phenotypic status. It can also be used to find out how many of each type of cell is present in a certain microenvironment, which is very useful for studying developmental biology [24]. The number of computer programs that can analyse bulk RNA-seq and scRNA-seq data is growing quickly [25, 26]. This makes it easier to find new biomarkers for COAD patients who do not have a good outlook, which is very useful for clinical use. In this study, we have built a COAD prognostic risk model using four immune function gene sets, with each having an enrichment score confirmed by several datasets. We have looked into how the model groups' immunological environments and expressions have varied from one another. Then, using single-cell data analysis, four tumor cell subsets with the four immune functions have been identified. The Cancer Genome Atlas (TCGA) dataset has been employed to combine Differentially Expressed Genes (DEGs) from these and other subsets to find genes linked to COAD prognosis. The Risk Score (RS) model has then been constructed to assess COAD prognosis using prognosis-related genes. The TCGA internal training set, the global set, and the Gene Expression Omnibus (GEO) external validation set have all confirmed the model’s good and stable prognosis evaluation efficiency. The relationship between the RS model and TME, immune function, genome variation, and immunotherapy has been investigated. The RS model we have developed has assisted in classifying the prognosis of COAD patients and been very useful in predicting the efficacy of immunotherapy and improving the prognosis of COAD patients.

## 2 MATERIALS AND METHODS

### 2.1 Expression Data Downloading and Preprocessing

The FPKM expression spectrum data from the TCGA's COAD dataset was converted to log2 (FPKM + 1). For survival analysis, an integrated clinical data resource for pan-cancer from TCGA to produce superior survival outcome analytics [27] was used. Simultaneously, the mutation data from this data were downloaded for genome change analysis.

Following their retrieval from the GEO database, the expression spectrum and survival data from the GSE29623, GSE39582, and GSE41258 datasets were analyzed as follows: to get rid of the samples, (1) Those samples that did not have clinical follow-up data were eliminated; (2) Those with less than 0 days, no survival status, and the unified survival time unit of days were removed; (3) The probe was changed into a gene symbol; (4) One probe matched more than one gene, so it was removed; and (5) The expression with multiple gene symbols was used as the median value. The aforementioned dataset was the GEO external validation set for the RS model. The immunotherapy dataset was then obtained, containing the expression profile and clinical details from the GSE176307 dataset.

We obtained the COAD single-cell dataset GSE146771_10X expression profile file and meta information file from the Tumour Immune Single Cell Hub database so that Seurat could use them to build single-cell objects.

### 2.2 Downloading Immune Function Gene Set

We obtained the gene sets pertaining to immune function through a literature reference [28], and the results have included 33 immune function gene sets.

### 2.3 Enrichment Analysis

The single sample Gene Set Enrichment Analysis (ssGSEA) algorithm from the R package GSVA was used to find out the gene set enrichment score (ICF pathway/KEGG/HALLMARK) of each cancer sample [29, 30]. This was done by looking at the gene expression of TCGA COAD samples. The algorithm first calculated each gene’s cumulative distribution density function in all samples. In this process, all samples were considered for analysis.

### 2.4 Single-cell Data Clustering

The single-cell dataset was analysed using the specialised single-cell transcriptome analysis tool. The primary stages in this analysis included the production of objects, standardisation of data, reduction of data dimensions, grouping, and identification of marker genes. Originally, the CreateSeuratObject() function in Seurat was employed to build a Seurat object. The minimum number of features and cells were both set to 200 and 3, respectively, at the same time. Following the initial stage of gene and cell filtering, we set the overexpression of doublet_cutoff = 0.15, set the number of counts to 300,40000, and set the number of features to 200,6000. The percentage of red blood cell readings was less than 20%, while the percentage of mitochondrial genes was less than 30%. The ArrowPlot() method was then used to obtain the principal component fraction for further investigation. Lastly, tumour cell subsets were identified using FindClusters(). After that, the FindAllMarkers() [31] function was used to set min.pct = 0.25 and logFC.threshold = 0.25 to find all marker genes of all subgroups. The threshold was set to avg_log2FC > 1 and *p*_val_adj < 0.05 to find the top markers of subgroups. We used the function FindMarkers() to look at the difference in expression between special ICF cell subsets and other cell subsets. The cutoff values were *p*_val_adj < 0.05 and avg_log2FC > 1. This helped us find the unique expression genes of these cell subsets. The Uniform Manifold Approximation and Projection (UMAP) is a well-known way to learn about reducing dimensions. It is based on the theoretical framework structure of Riemannian geometry generation data. When processing high-throughput and high-dimensional data, such as single cells, it maintains more global structures, performs better operationally, and is more scalable. Lastly, the landscape distribution and tumour cell gene expression were visualised using the DotPlot(), DoHeatmap(), and VlnPlot() tools [32-33]. 

### 2.5 Cellular Communication

Using scRNA-seq data, the R software CellChat [34] detects cell-to-cell communication. In CellChatDB [35], one can find polymers, coenzyme factors, and ligand-receptor interactions that have been proven in human and mouse studies. Additionally, distinct ligand libraries exist in humans that are capable of finely illustrating the intercellular interaction from a particular angle. This analysis employed single-cell data to infer cell communication from all CellChatDB.human databases. 

### 2.6 Genomic Signature Nucleotide Variation (SNV) Analysis

A summary map of mutation information for high- and low-risk groups was made using the plotmafSummary() function of the R package maftools. This was based on the maf file that contained the somatic mutation detection results from the TCGA-COAD queue. Next, a waterfall map was created using the oncoplot() method [36] to display the variations in SNV mutations among the various groups. 

### 2.7 The Role of Tumour Immune Dysfunction and Exclusion (TIDE) in Immune Response Prediction

The TIDE score algorithm is based on the tumor's expression profile before treatment and scores several published transcriptome biomarkers to predict patients' immune responses. The TIDE score models how the immune system can get around a tumour by using different numbers of cytotoxic T cells that can enter the tumour and kill cells. It does this by combining T-cell malfunction and elimination properties. The TIDE score stands out from the other biomarkers. Regardless of the extent of tumor infiltration by cytotoxic T cells, its efficiency remains constant. 

### 2.8 Sample Collection

After removing the COAD samples and surrounding tissues from 12 patients, they were promptly frozen in liquid nitrogen and kept at -80°C. Anti-tumour therapies were not given to any of the COAD patients prior to surgery. After being fully informed, participants in the trial gave their informed consent, as did the patients and their families. The Xishan People's Hospital’s ethics committee gave its approval for this study.

### 2.9 qPCR Analysis

The TRIzol reagent, manufactured by Invitrogen (Thermo Scientific, Shanghai, China), was employed to extract the entirety of RNA from the CRC tissue specimens. The QuantiTect Reverse Transcription Kit, manufactured by QIAGEN in Valencia, CA, USA, was employed to perform the reverse transcription of RNA into complementary DNA (cDNA). For the real-time PCR investigations, SYBR-Green (Takara, Otsu, Shiga, Japan) was utilised, with the levels being normalised to the GAPDH level.

### 2.10 Immunohistochemical Staining

Tissues fixed in paraffin were immunostained for the proteins ASAH1, CMC1, and S1PR5. The slides underwent deparaffinization, drying, and rehydration. Following an overnight immersion in 3% hydrogen peroxide, the slides were tagged with antibodies. The anti-S1PR5 (1:200), anti-CMC1 (1:200), and anti-ASAH1 (1:200) antibodies were purchased from Abcam (Cambridge, UK). 

### 2.11 Cell Culture and Transfection

The human CRC cell lines SW480 and SW620 were supplied by the ATCC Cell Bank of the United States. Cells labelled as SW480 and SW620 were cultured in Leibovitz's L-15 media (Sigma, Beijing, China) at a temperature of 37°C in an incubator without CO_2_. The medium was enriched with 10% Foetal Bovine Serum (FBS) obtained from Gibco, Grand Island, NY, USA, 2 mmol/l glutamine, 100 U/ml penicillin, and 100 μg/ml streptomycin were obtained from Thermo Scientific, Waltham, MA, USA.

### 2.12 EDU Assay

For cell seeding, 5×103 cells per 96-well plate were used. We put the 96-well plates in an incubator at 37°C with 5% CO_2_ for two hours after adding 20 μM Edu labelling medium (KeyGENBioTECH, Nanjing, China). This was done 48 hours after transfection. Next, in accordance with the manufacturer's instructions, the BeyoClickTM EdU-488 kit (No. C0071S, Beytime, China) was used to perform the EDU assay.

### 2.13 Statistical Analysis

In the statistical mapping, the Kruskal-Wallis test was used to assess disparities among many groups of samples, while the Wilcoxon test was used to investigate differences between two groups of samples. In this instance, "ns" represents a *p*-value more than 0.05, whereas "*", "**", and "***" represent *p*-values less than or equal to 0.05, 0.01, 0.001, and less than 0.0001, respectively.

## 3 RESULTS

### 3.1 Construction and Evaluation of ICF-related RiskScore (ICFRS)

#### 3.1.1 Constructing ICFRS by TCGA Dataset

First, a differential analysis of the enrichment scores of immune function gene sets was conducted to determine the difference in immune function between tumor and normal tissues, and 20 differentially enriched immune function gene sets were identified. Fig. (**1A**) depicts the results; 5 gene sets had a higher enrichment score in tumor tissues, while 15 gene sets had a higher enrichment score in normal tissues. Then, we selected 7/10 of the TCGA dataset as the training set. Lasso analysis was employed to construct COAD ICFRS based on the enrichment scores of these 20 gene sets. Figs. (**1B**, **C**) show the results; finally, four immune function gene sets related to prognosis were found. These included innate immunity, inhibitory ligand, chemokine gene ontology, and TGF-β response. Fig. (**1D**) displays the survival curves of the high- and low-ICFRS groups. The low-risk group demonstrates a significantly more favorable prognosis compared to the high-risk group. Fig. (**1E**) displays the RS curve and scatter chart depicting the distribution of survival time and survival status in the training set. On the other hand, Fig. (**1F**) illustrates the disparity in enrichment score distribution between the high- and low-risk groups in the training set for the four ICF gene sets. The TCGA ensemble was used for validation to confirm the model’s prognostic effectiveness. The results are shown in Figs. (**1G**-**I**); there were significant differences in the survival of high- and low-risk groups. Low-risk groups’ prognosis was also better than high-risk groups. The H and I charts show that the distribution of RS and survival status and the distribution of ICF enrichment scores in the TCGA ensemble’s high- and low-risk groups followed the same trend as the TCGA training set.

#### 3.1.2 Verification and Evaluation of ICFRS

In order to corroborate the prognostic effectiveness of ICFRS, the model was validated using two GEO external validation sets. The findings are displayed in Figs. (**2A**, **B**). The survival curves of the two datasets for the high- and low-ICFRS groups differed significantly from one another. The different patterns of ICF enrichment scores in high-risk and low-risk groups, as shown in Figs. (**2C**, **D**), have been found to be in line with the TCGA dataset. To compare the immune function of the high- and low-risk groups, a box chart was created to display the differences in enrichment scores for these 33 immune pathways. Fig. (**2E**) presents the findings. Low-risk groups' immune function enrichment scores were generally higher than those of high-risk groups, suggesting that most of these immune functions are beneficial to COAD prognosis. The enrichment scores of 28 immune function gene sets differed significantly between the high- and low-ICFRS groups.

After that, differential expression analysis was performed on the high- and low-risk groups of the TCGA ensemble, and a volcanic map was created. Fig. (**2F**) presents the findings. 104 genes were found to differ significantly. We performed enrichment analyses of GO and KEGG pathways based on DEGs to find out how the high- and low-ICFRS groups were different in how they worked. We then created a bubble diagram showing the top 10 significant enrichment pathways. The results are shown in Fig. (**2G**); DEGs were mostly rich in functions and pathways related to chemokines, ligand-receptor signaling, antibacterial immune response, and leukocytes.

### 3.2 Identification and Characterization of Prognosis-related ICF Cell Populations

#### 3.2.1 Single-cell Tumor Landscape

Through cell classification and subtype discovery, single-cell transcriptome analysis could deeply study and explore the cell function in tumor tissue. It can also be applied in therapeutic domains, like immunology, illness categorization, bone marrow transplantation, and tumor cell heterogeneity investigation.

This single-cell dataset contained cells from three source sites of 10 patients: tumor (cancer tissue), NAT (Normal tissues Adjacent to the Tumour), and Peripheral Blood Mononuclear Cell (PBMC). First, the cells in the tumor and NAT sites were selected. The difference in cell distribution between the tumor and normal tissues was assessed.

We demonstrated NAT sites using the annotation results from the original dataset. The results are shown in Fig. (**3A**). The difference in cell group distribution between the tumor and NAT sites was significant. Tumors had a higher proportion of T cells, M1 cells, and monocytes than normal tissues. All the tumor cells were selected for subsequent analysis. First, Seurat objects were generated. Following the initial dimension reduction analysis (principal components analysis), the top 10 PCs were chosen for further dimension reduction and analysis. As shown in Fig. (**3B**), the population distribution of single cells was then shown using UMAP dimension reduction and the single cell dataset's cell group annotation results. Later, to accurately cluster and discover cell subsets, a cluster with a resolution between 0.1 and 1.0 was drawn, as shown in Fig. (**3C**). After the resolution exceeded 0.4, frequent leaps among cell subsets begin to occur, indicating that excessive clustering may occur, so we chose 0.4 as the resolution for cell subgroup discovery. At 0.4 resolution, SingleR was used to annotate the cell subgroups. The results are shown in Fig. (**3D**). The 12 cell subgroups were identified, and their annotation results have been found to be consistent with those of the original dataset. The cumulative distribution histogram was subsequently drawn. Fig. (**3E**) shows the difference in cell types between different individuals, indicating the heterogeneity of TME to be very strong. Simultaneously, Fig. (**3F**) shows the difference in cell type distribution across Tumor Node Metastasis (TNM) stages. The proportion of B cells_1, CD8+T cells_0, and T cells_4 was higher in patients with more advanced TNM stages. As shown in Fig. (**3G**), each subgroup's TOP5 marker gene was selected, and a violin diagram was drawn to show the expression characteristics of these 12 cell subgroups.

#### 3.2.2 Identification of Prognosis-related ICF Cell Subsets

Top markers were chosen for each of the 12 subgroups so that we could look into how different ICF-related gene expression affects the prognosis of these groups. Next, a bubble diagram was made to show how each cell subgroup's expression of top markers corresponded to the prognosis for ICF. Fig. (**4A**) presents the findings. Every subgroup had prognostic ICF-related gene expression that we could see, with subgroups 6 and 7 exhibiting the highest expression specificity. To look at how these 12 subsets were enriched in the four ICF gene sets related to prognosis, the average expression value of the genes in each subgroup of the single-cell dataset was found, and ssGSEA enrichment was checked.

**Fig. (4):**
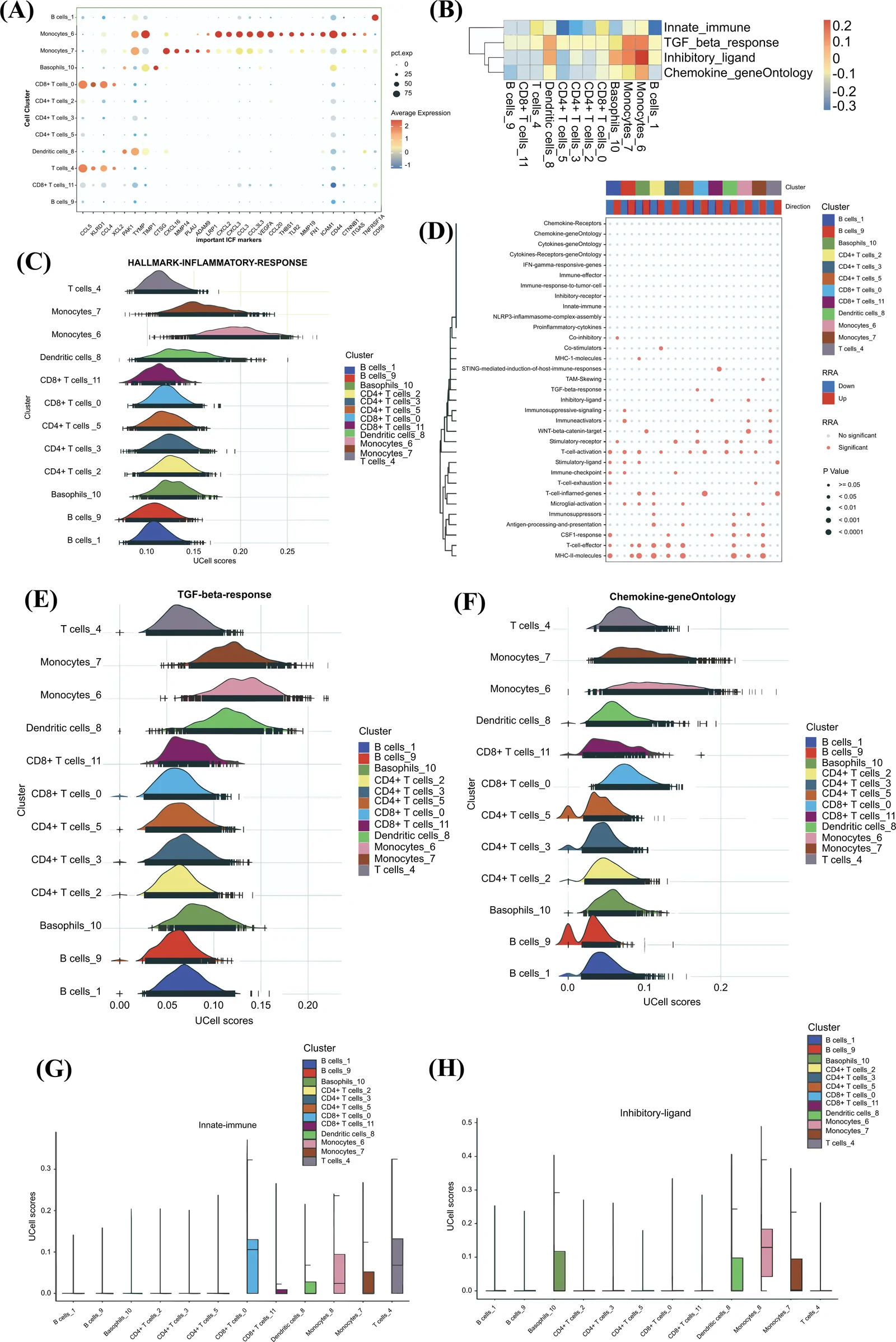
**A**) Recognition results of cell subsets with ICF prognosis. The bubble diagram illustrates the expression of top markers associated with ICF prognosis in each cell subgroup. The redder the color, the higher the average expression value. The dot's size represents the number of expressed cells. **B**) The ICF irGSEA enrichment score heatmap, which is based on the average expression of subgroup genes. **C**) The enrichment mountain map of the HALLMARK proinflammatory response pathway, in which the abscissa represents the enrichment score and the ordinate represents the distribution of samples in different cell subsets at this score. **D**) A bubble diagram illustrating the irGSEA enrichment results of 33 ICF gene sets. (**E**-**H**) The mountain map and half violin map present the irGSEA enrichment results of four ICF prognosis gene sets.

The results showed that subgroups 6 and 7 of tumor cells had a relatively high enrichment score for Chemokine_geneOntology, TGF_beta_Response, and inhibitory_ligand of subgroups. The enrichment score for subgroups 0 and 4 of the Innate_immune was higher (Fig. **4B**). Using the enrichment analysis algorithm on the single-cell dataset, the enrichment scores of each subgroup's HALLMARK pathway were calculated. It was found that subgroups 6 and 7 had the highest enrichment scores for proinflammatory responses (Fig. **4C**). Similarly, enrichment analysis was conducted on the immune function gene set, and a bubble diagram of enrichment results was drawn. As shown in Fig. (**4D**), the gene set with the lowest enrichment results was the most significant. We discovered, when looking at these four ICFs together, the prognosis-related enrichment scores to not be as strong as those of other ICFs. This may be likely because their gene expression was not all the same. The enrichment mountain map and half violin map of these four ICF genes were, respectively, drawn based on enrichment results. Figs. (**4E**, **F**) show the enrichment mountain map of the Chemokine_geneOntology and TGF_beta_response gene sets. Subgroups 6 and 7 had the highest enrichment scores, while subgroups 0 and 4 had high enrichment scores. Due to the low enrichment scores of Inhabitory_ligand and Innate_immune, the half-violin chart (Figs. **4G**, **H**) was used to better show the enrichment differences among the subgroups. The enrichment scores of Innate_immune in subgroups 0 and 4 were higher than those of Inhabitory_ligand in subgroups 6 and 7.

#### 3.2.3 Cellular Communication

The communication among cell subsets was characterized using SingleR annotation results and expression information from a single-cell dataset. First, a cellular communication network diagram was created using the R package’s built-in function. The intercellular communication network is a weighted digraph made up of significant ligand-receptor pairs among interacting cell groups. It shows how many ligand-receptor connections there are between different types of cells. The outcomes are displayed in Fig. (**5A**); the thickness of the line connecting the two subgroups indicates the degree of frontal communication between them, while the dot size indicates the total number of cells in this subgroup. The number of links in the cell communication net indicates the level of communication between the cells. The number of links between each cell was then visualized using a heatmap. According to Fig. (**5B**), there was close communication between subgroups 6 and 7 and other subgroups, and the communication intensity between subgroups 0 and 4 was also relatively high. In cell communication, we may determine the strength of various cell subgroups as signal senders and receivers by using the out-degree and in-degree in-network analysis. Fig. (**5C**) shows that 6 and 7 cell subgroups had the most signal senders, while 0 and 4 subgroups had the most signal receivers. Finally, to investigate how multiple cell groups and signal pathways interact, a pattern recognition method was used to identify the global communication pattern and key signals of each subgroup, and a heatmap was created. Fig. (**5D**) shows which signals contributed the most to the outgoing or incoming signals of each cell group and the centrality_scores diagrams of each signal pathway are shown in the attachment. Cell communication analysis revealed that cell communication among the four prognosis ICF subsets reflected synergism between the four cell subsets.

#### 3.2.4 Functional Identification of Prognosis-related ICF Cell Populations

Cell subgroups 0, 4, 6, and 7 were identified as having ICF prognostic function based on the preceding analysis. To find out what these four subgroups were used for, differential expression analysis was conducted on them compared to other non-ICF subgroups. Then, based on the specific marker gene of these groups, fgsea was used in a KEGG pathway enrichment study to determine the shared roles of these cells and common enrichment pathways were selected to draw the Normalized Enrichment Score (NES) value heatmap. The results are shown in Fig. (**6A**). Based on the sequence of the log2FC value of the marker gene in each subgroup, the HALLMARK pathway was GSEA enriched. The top 10 pathways with enrichment significance were thus obtained. The findings are shown in Figs. (**6B**-**E**). The main ways that co-enrichment occurred were through the interferon response, allograft rejection, TNFA signal, and other pathways.

### 3.3 Identification and Verification of Prognosis ICF Signature

#### 3.3.1 Recognition of ICF Signature in the Prognosis of COAD

To look into how prognosis-related ICF can help diagnose COAD, the unique genes of four groups of prognosis-related ICF cells were put together to make 462 ICF markers. After that, 13 genes linked to the prognosis of COAD were found, univariate Cox analysis was revealed (*p* < 0.01), and ICF indicators were built. Fig. (**7A**) displays the forest map illustrating the findings of the univariate study. Subsequently, a random sampling method was employed to select 304 out of 435 samples from the TCGA-COAD ensemble, representing 7/10 of the total. Figs. (**7B**-**D**) shows a selection of 10 prognostic-related signatures. These signatures were identified after removing redundant genes in the training set using Lasso linear regression with a seed value of 1,110. The K-M survival curve of the TCGA ensemble was generated by utilizing the median of each gene expression as the threshold value to divide the samples into high and low groups. The results are presented in Fig. (**7E**), showing distinct differences in the K-M curves for six genes.

#### 3.3.2 Effectiveness of the TCGA Internal Validation Model

We aimed to evaluate the influence of the model's score, derived from 10 specific attributes, on the total survival duration in the training dataset. Initially, the K-M curve was plotted utilizing the RS median as the critical criterion. The samples were grouped into risk groups, with certain samples designated as high-risk and others as low-risk. The results are presented in Fig. (**8A**). The samples from the high-risk group exhibited a more unfavorable prognosis, whereas the Kaplan-Meier curve of the low-risk group demonstrated a p-value of less than 0.01, indicating a substantial disparity between the two groups. The Receiver Operating Characteristic (ROC) curve of the prognosis signature was generated using the risk model, as illustrated in Fig. (**8B**). The Area Under the Curve (AUC) values for 1, 3, and 5 years were 0.709, 0.758, and 0.780, respectively. These values indicated the model's ability to predict scores as highly effective. We also created scatter plots for survival time, survival state, and sample RS. By combining these two scatter plots, we could reveal the relationship between survival and score. The findings are presented in Figs. (**8C**-**E**), which depict the portrayal of specific genes in the training set's high-risk and low-risk groups.

The entire TCGA-COAD dataset was utilized to evaluate the prediction capability of RS for overall survival. The samples were classified into high-risk and low-risk categories using the same criteria as the one used in the TCGA training set. As shown in Supplementary Fig. (**1A**), the high-risk group had a worse prognosis, and this difference in prognosis was statistically significant. In Supplementary Fig. (**1B**), the AUC values for the overall concentration of TCGA could be seen to be 0.679, 0.696, and 0.684 for 1, 3, and 5 years, respectively. Supplementary Fig. (**1C**) displays a scatter chart illustrating the RSs (Risk Scores) of the samples in the overall concentration. Supplementary Fig. (**1D**), on the other hand, presents a scatter chart depicting the distribution of the RSs in the samples based on survival time and survival state. Supplementary Fig. (**1E**) shows the heatmap of the expression of the model gene in the entire dataset. The TCGA ensemble's validation results demonstrated that the model score consistently and positively affected the survival prediction.

#### 3.3.3 Verifying the Robustness of the Model with External Datasets

The external dataset GSE41258 was used to perform the same analysis and verification in order to further confirm the reliability of the model score in predicting the overall survival time of COAD patients. The results are displayed in Supplementary Fig. (**2**). The prognosis for samples from high-risk groups was worse, and the Kaplan-Meier curve of high-risk groups (*p* < 0.05) showed a statistically significant difference in prognosis between the two groups; the AUC values for 1, 3, and 5 years were 0.657, 0.670, and 0.652, respectively. These values suggest the model's predictive accuracy to be high. Concurrently, the scatter chart depicting survival time and survival state as well as the scatter chart illustrating sample RS were generated. By merging these two scatter charts, we could uncover the correlation between survival and score. The outcomes illustrated the expression of model genes in groups categorised as high-risk and low-risk.

Concurrently, we conducted the same analysis and verification on an additional external dataset, GSE29623. The results are displayed in Supplementary Fig. (**3A**), which demonstrates that the prognosis of samples from high-risk groups was more unfavorable. The K-M curve of high-risk groups (*p* < 0.05) revealed a substantial disparity in prognosis between the two groups. In Supplementary Fig. (**3B**), the Area Under the Curve (AUC) values for 1, 3, and 5 years can be found to be 0.766, 0.658, and 0.647, respectively. These values indicate the model's predictive effectiveness to be good. Scatter plots depicting the relationship between survival time and survival state, as well as the scatter plot illustrating the sample RS, have been created. By merging the scatter charts, the correlation between survival and risk score could be elucidated. The results are depicted in Supplementary Fig. (**3C**, **D**), whereas Supplementary Fig. (**3E**) illustrates the expression of model genes in groups categorised as high-risk and low-risk.

### 3.4 Correlation between the Risk Model and Certain Tumor Features

#### 3.4.1 Risk Model Related to Clinical Characteristics of COAD

**Fig. (9):**
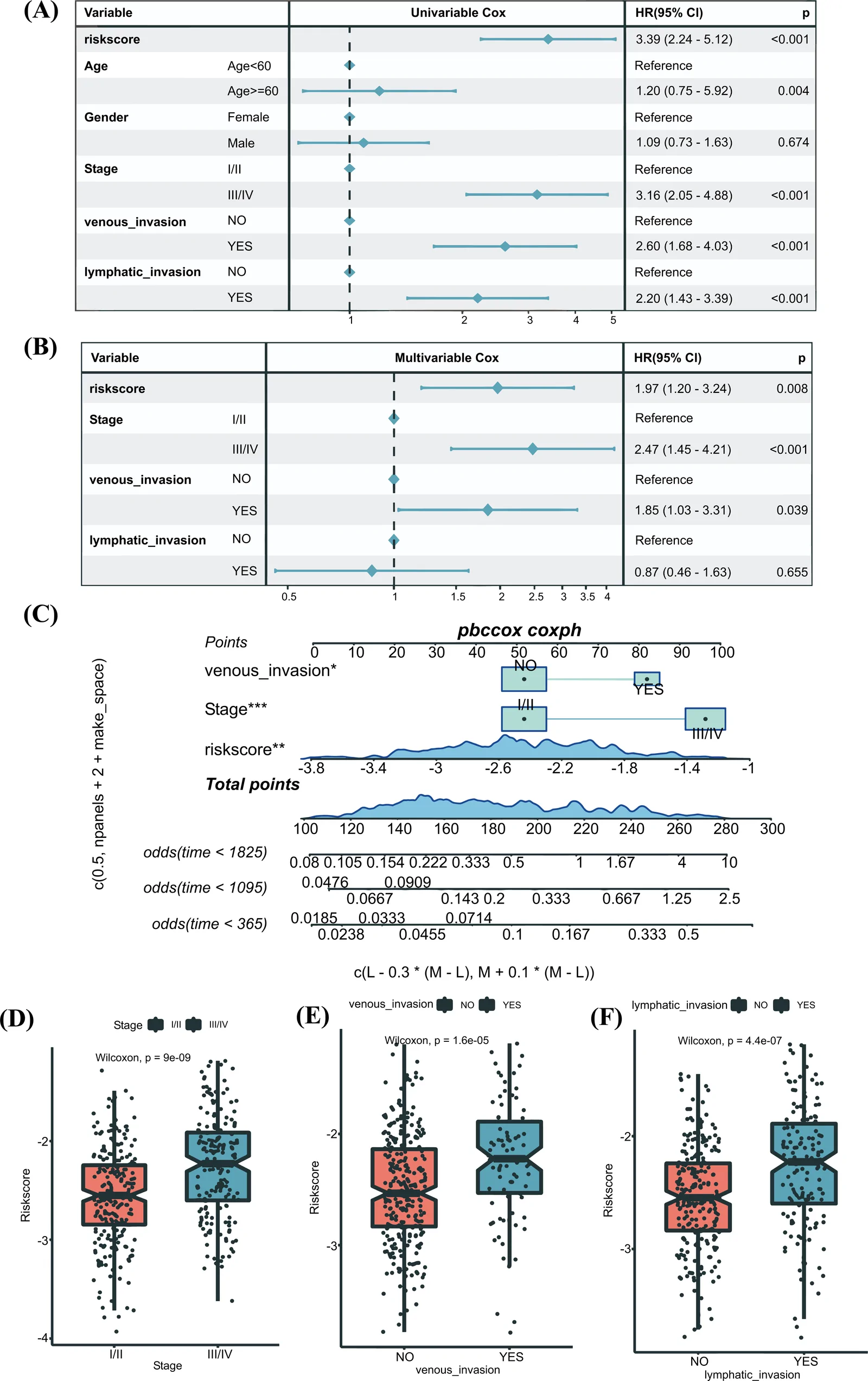
Model score independence in clinical characteristics. **A**) The forest map displays the results of the univariate Cox analysis of clinical factors in the TCGA cohort. **B**) The forest map displays the results of the multivariate Cox analysis of independent factors in the univariate analysis. **C**) The prediction model's histogram illustrates the clinical factor's contribution to the outcome event. (**D**-**F**) The total points indicate the sum of the corresponding single-item scores after accounting for all variables, and the bottom three lines indicate the corresponding 5/3/1 year survival probability of each value point. Different colors represent different groups, and the *p*-value represents the significant difference.

This analysis has integrated the variables of age, gender, stage, venous invasion (nerve invasion), and lymphocytic infiltration. This study has used demographic and clinical data from patients with COAD, including age, gender, stage, venous invasion (nerve invasion), and lymphocytic invasion (lymph invasion). The data have been analysed using univariate and multivariate Cox regression analyses to investigate whether RS can serve as an independent predictive factor. The results are presented in Figs. (**9A**, **B**); in both the univariate and multivariate Cox regression analyses, the RS, stage, and venous invasion of the model were very different from the reference, showing them to be separate factors that can help predict the outcome. Another thing involved in making the nomogram (Fig. **9C**) was survival time and survival status, along with age and the clinical pathological stage of clinical indicators; RS and pathological stage were notable clinical factors. The distribution of RS among clinical feature groups was then investigated. The findings are presented in Figs. (**9D**-**F**), which reveal significant variations in RS throughout different stages, including venous invasion and lymphatic invasion. This suggests that several clinical characteristics of COAD are linked to RS.

#### 3.4.2 Models Related to TME

**Fig. (10):**
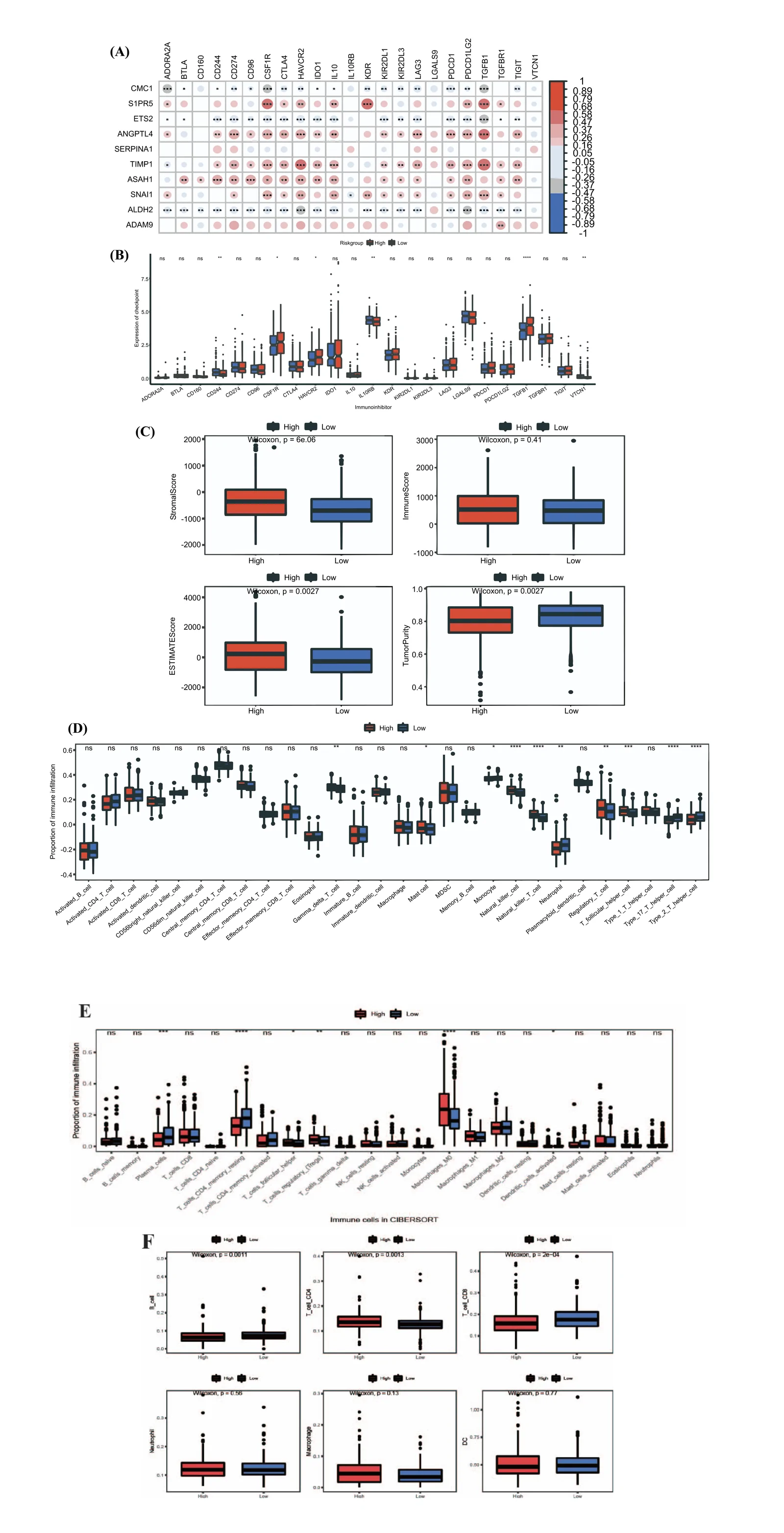
The TME difference of the model grouping. (**A**) Heatmap depicting the correlation coefficient between model gene expression and immune checkpoint. It shows the level of correlation and the significance of the spot colour. **B**) The box graph shows differences in inhibitor expression at 23 immune checkpoints between high-risk and low-risk groups. The high-risk group is shown in red, and the low-risk group is shown in blue. **C**) The difference box chart of matrix score, immune score, ESTIMATE score, and tumour purity. (**D**-**F**) The difference box chart shows the percentage of immune infiltrating cells found in high-risk groups using the ssGSEA, CIBERSORT, and TIMER algorithms.

Immune checkpoints are a collection of molecules found in immune cells that have the ability to control the degree of immunological activation. They have a significant impact on human autoimmune disease development. In order to examine the variations in immune checkpoint expression across different models, we initially computed the correlation between the expression of 10 genes and the expression of immune checkpoints. Subsequently, a correlation heatmap was generated. The findings are presented in Fig. (**10A**), which indicate a strong correlation between the expression of various immunological checkpoints and the expression of model genes. Ultimately, the levels of 23 Immunological Checkpoint Blockades (ICBs) were compared between the groups classified as high-risk and low-risk. Fig. (**10B**) displays the box graph's results, which reveal differences in the expression of six ICBs between the high-risk and low-risk groups. This study has looked at the difference in the Tumour Microenvironment (TME) between high-risk and low-risk groups based on the percentage of immune cells that invaded the tumour. The box chart illustrates the disparities in the four ESTIMATE scores among high-risk categories. The high-risk group's matrix score, immunological score, and estimate score were significantly higher than the low-risk group, although the tumor purity was lower (Fig. **10C**). Fig. (**10D**) presents a box graph illustrating the proportion difference of immune infiltrating cells. This evaluation was conducted using the SSGA algorithm for 28 different types of immune cells, with 10 of them showing significant differences. Based on the COBERSORT algorithm, Fig. (**10E**) shows how the percentage of infiltration changed for 22 different types of immune cells across different subtypes. Notably, there were large disparities in the infiltration proportion of six specific cell types. The annex displays the results of the computations performed by the other two algorithms. Fig. (**10F**) presents a box chart illustrating the disparity in the proportion of immune cells across six categories, as determined by the TIMER algorithm. The figure reveals a large variation in the levels of immunological infiltration by B and T cells across high-risk groups. The xCell algorithm's immune infiltration proportion results can be seen in the appendix.

#### 3.4.3 Risk Models Associated with Tumor Genome Mutations

Gene mutation can facilitate and contribute to the development of cancer or collaborate to propel the malignant proliferation of cancer. Understanding genome-wide mutations is critical for developing tumor-specific medications and novel tumor treatments. First, the overall mutation in the TCGA-COAD dataset is displayed in combination with clinical grouping information. Figs. (**11A**, **B**) provide a summary map of mutation information and a top 20 waterfall map of mutation frequency for high-risk group samples, while Figs. (**11C**, **D**) present a summary map of mutation information and a top 20 waterfall map of mutation frequency for low-risk group samples, visualising the allocation of genetic variants among high-risk groups and samples with distinct clinical characteristics. The APC gene exhibited the highest frequency of COAD mutations, whereas the TP53 gene demonstrated a significantly greater mutation frequency in the high-risk group compared to the low-risk group, indicating that mutation of this gene may be detrimental to COAD prognosis.

#### 3.4.4 Gene Set Variant Enrichment Analysis

The study has investigated the disparity in pathway enrichment among high-risk groups by utilizing the immune function gene set of COAD samples and the HALLMARK pathway enrichment score. This score has been found to be useful for investigating the correlation between cancer characteristic pathways and prognosis. Fig. (**12A**) shows that the enrichment scores of different gene sets related to immune function were very different between the model groups. These included three gene sets linked to prognostic ICF. Similarly, a heatmap of the enrichment scores was generated to compare the high-risk and low-risk groups. For this analysis, the samples' HALLMARK enrichment values were used. Statistical tests were then applied to determine the significance of the difference. Fig. (**12B**) displays the results. There were substantial disparities in the enrichment ratings of 25 channels between the high- and low-risk groups. Concurrently, the relationship between model gene expression and the score indicating pathway enrichment was computed. The results are displayed in Figs. (**12C**, **D**). The expression of the TIMP1 and ANGPTL4 genes was highly correlated with the enrichment scores of several pathways.

### 3.5 Risk Model to Predict the Therapeutic Effect on Patients

#### 3.5.1 Risk Model Assessment of Chemotherapeutic Drug Resistance

Using TCGA-COAD expression data, predictions were made for 138 medications' IC_50_ values in the GDSC database. Several analyses that used model grouping outcomes and IC_50_ values chose drugs that had big differences in IC_50_ values between high-risk groups. Drugs with notable differences were chosen to compute the association between the expression of model genes and the drugs' IC_50_ values. Fig. (**13A**) demonstrates the outcomes. The TIMP1 gene expression had a significant negative correlation with the medication's IC_50_ value. Fig. (**13B**) depicts the distinct distribution of IC_50_ values for sensitive medicines among high-risk categories. The high-risk group exhibited greater susceptibility to 11 medications, whereas the low-risk group demonstrated heightened sensitivity to 44 pharmaceuticals. A box plot was constructed to illustrate the disparity in drug sensitivity between high-risk and low-risk groups, based on IC_50_ values. Additionally, three medicines that exhibited greater sensitivity in both high-risk and low-risk groups were chosen for presentation. The results are displayed in Figs. (**13C**-**H**). The high-risk group exhibited greater susceptibility to medications presented in Figs. (**13C**-**E**), while the low-risk group had heightened sensitivity to drugs shown in Fig. (**14**). The annex contained the remaining pharmacological findings.

#### 3.5.2 Risk Model to Predict the Efficacy of Immunotherapy

The study's goal was to find out if model genes can be used as signs of how well immunotherapy is working and to see how well tumour RS can predict how well immunotherapy will work for patients. Initially, the immunotherapy datasets were segregated into groups of high- and low-risk based on the RS model. Subsequently, a Kaplan-Meier curve was plotted to analyse and evaluate the disparities in the survival rates. Fig. (**14A**) illustrates the outcomes. Within the immunotherapy cohort, the prognosis for high-risk individuals was notably distinct and unfavorable. The difference in RSs between the groups with and without response was then plotted on a violin chart, as shown in Fig. (**14B**). The group's RS was higher without a response. Meanwhile, as depicted in Fig. (**14C**), the cumulative distribution histogram of immunotherapy response was plotted for the high-risk group. The group with the lowest risk exhibited a higher rate of objective reaction. The sample's immune response in the TCGA-COAD dataset was then predicted using TIDE online analysis, and a violin chart of the difference in RS distribution between the groups with and without reaction was drawn. The results are displayed in Fig. (**14D**). The reaction-free group had a higher reaction selectivity. Additionally, a histogram was created to visually display any notable disparity in immune response among the various model groups. The results are displayed in Fig. (**14E**). The percentage of individuals who responded positively in the low-risk group was much higher than that in the high-risk group. The Immunophenoscore (IPS) is a tool that can assess the immunogenicity of tumours and forecast their reaction to immunotherapy. The IPS scores of four groups were looked at to guess how the immune response would change in the model groups for high-risk groups. The groups were ips_ctla4_neg_pd1_neg, ips_ctla4_pos_ pd1_neg, ips_ctla4_neg_pd1_pos, and ips_ctla4_pos_ pd1_pos. The results are displayed in Figs. (**14F**, **G**). The IPS scores in low-risk groups exhibited a statistically significant increase compared to high-risk groups, suggesting the patients in low-risk groups to have a greater likelihood of experiencing positive outcomes following immunotherapy. The aforementioned findings suggest that individuals in the low-risk group were more inclined to experience positive outcomes after immunotherapy.

### 3.6 Expression and Function Analysis of the Unreported Model Genes S1PR5, CMC1, and ASAH1 in COAD

**Fig. (15):**
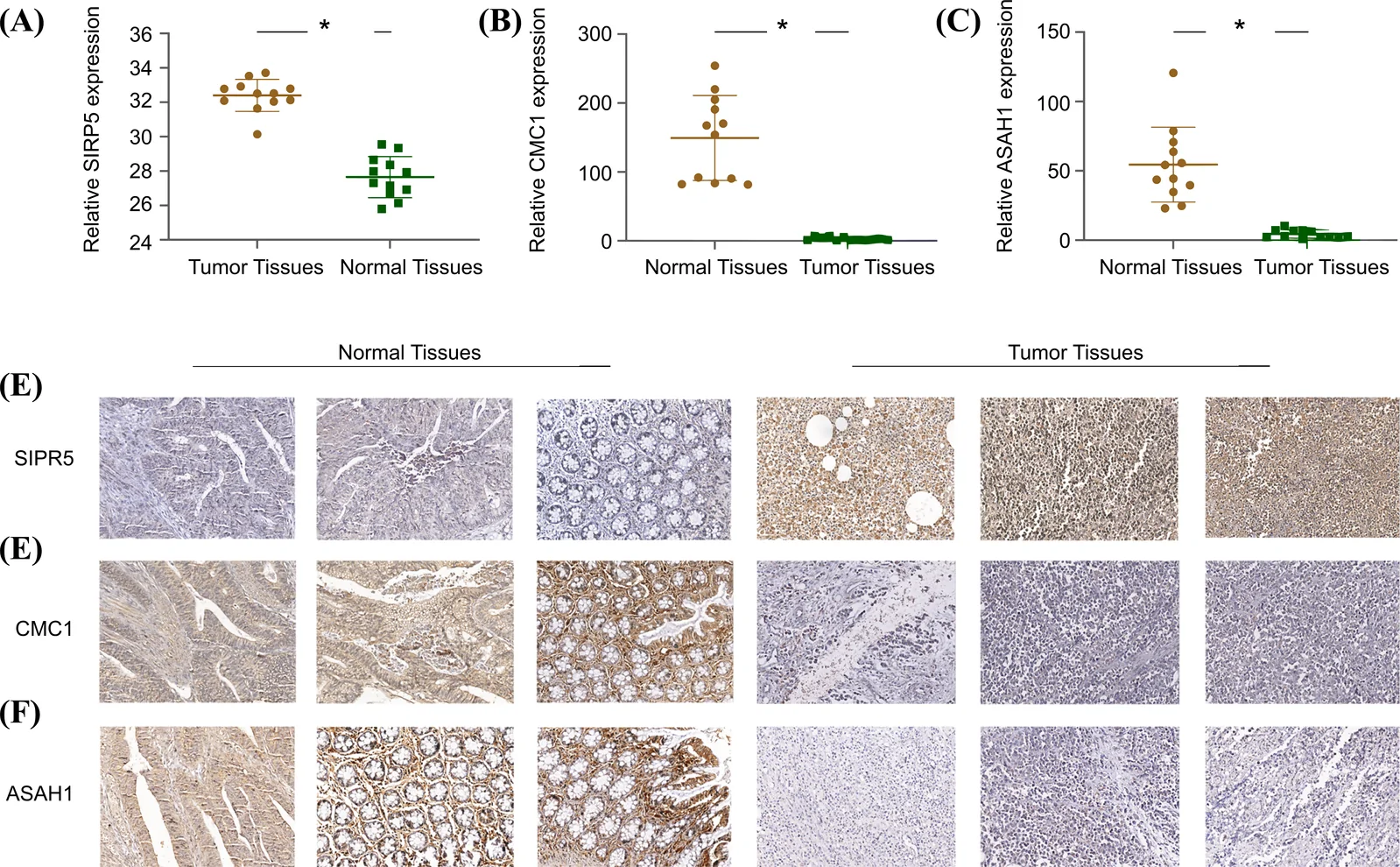
Expression of unreported model genes in COAD. (**A**-**C**): The qPCR analysis showed that the expression of S1PR5 was high and the expression of CMC1 and ASAH1 was low in COAD tissues. (**D**-**F**): The immunohistochemistry analysis also showed that the expression of S1PR5 was high and the expression of CMC1 and ASAH1 was low in COAD tissues.

Furthermore, we used quantitative Polymerase Chain Reaction (qPCR) and Immunohistochemistry (IHC) research to examine the expression of previously unknown model genes (S1PR5, CMC1, and ASAH1) in clinical samples obtained from patients diagnosed with Colorectal Adenocarcinoma (COAD). Both qPCR (Figs. **15A**-**C**) and immunohistochemistry (Figs. **15D**-**F**) showed that in COAD tissues, the expression of S1PR5 was greatly increased, while the expression of CMC1 and ASAH1 was greatly decreased.

**Fig. (16):**
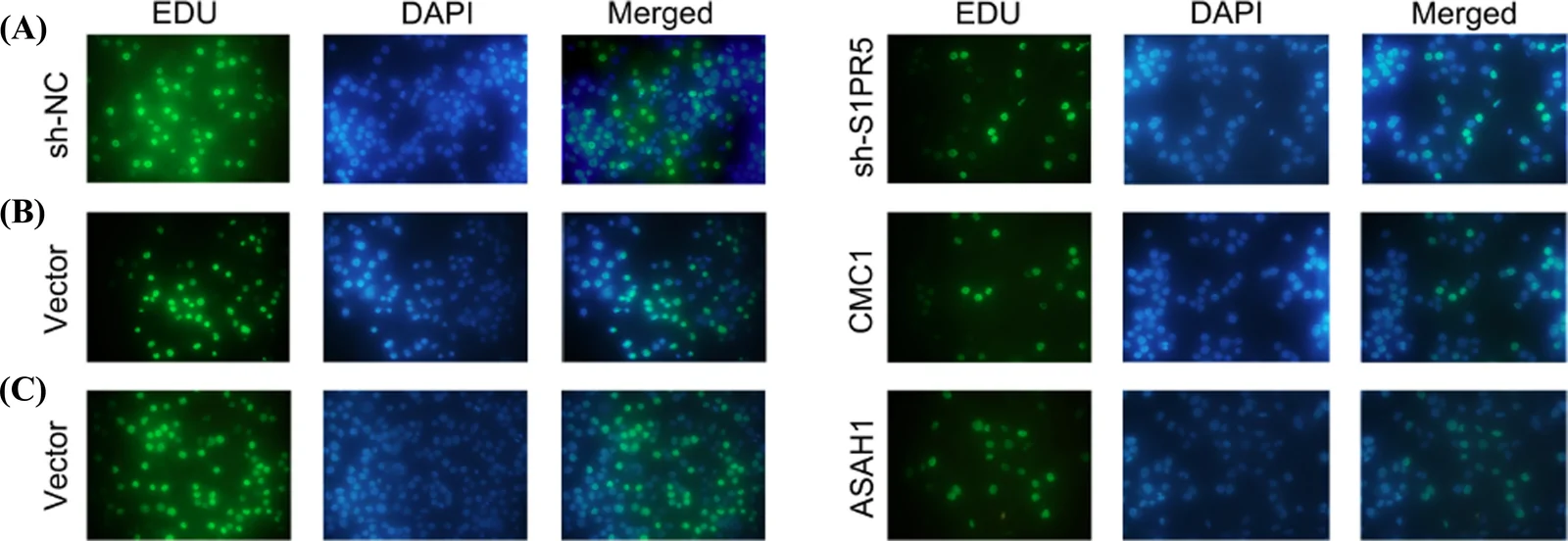
The function analysis of the unreported model genes S1PR5, CMC1, and ASAH1 in COAD. (**A**) The inhibition of S1PR5 expression inhibited the proliferation ability of SW620 cells *in vitro*. This resulted in tumorigenicity *in vivo*. (**B**, **C**) On the other hand, the overexpression of CMC1 and ASAH1 promoted the proliferation ability of SW480 cells *in vitro* and the tumorigenicity *in vivo*.

To find out what functions S1PR5, CMC1, and ASAH1 perform in colorectal cancer cells, an EDU assay and an orthotopic colorectal tumour model were used to test the regulatory roles of these model genes that were not known before. To make interference cell lines of S1PR5 (sh-S1PR5) in SW620 cells, the lentiviral expression vectors pLKO.1-NC-GFP and pLKO.1-S1PR5-GFP were used. The reference group consisted of SW620 cells that were transfected with lentiviral expression vectors (pLVX-IRES-Neo-3xFlag, pLVX-CMC1-IRES-Neo-3xFlag, or pLVX-ASAH1-IRES-Neo-3xFlag) to increase the expression of CMC1 (vector and CMC1) or ASAH1 (vector and ASAH1) in the SW480 cell line. The control group consisted of SW480 cells that were transfected with the vector. The findings showed that stopping S1PR5 from being expressed made it harder for SW620 cells to grow and divide in a lab setting that was under control (Fig. **16A**-**C**). The growth of SW480 cells in a lab setting, on the other hand, was improved by increasing the expression of CMC1 and ASAH1.

## 4 DISCUSSION

Tumour-infiltrating immune cells have been shown to have clinical significance in various human cancers [37]. Blocking the immune checkpoint is an effective and long-lasting treatment for different solid tumours [38-40]. Increasing evidence indicates the impact of TME on COAD tumorigenesis, prognosis, and metastasis [41-44], and there is an increasing number of immune prognosis models of COAD [45-47]. However, there are no appropriate features for systematically evaluating TME based on immune-related genes and predicting patients’ responses to immunotherapy in COAD. 

This study has identified four synergistic prognostic ICF gene sets in the TCGA-COAD cohort. The single-cell dataset was then analyzed to identify prognosis-related ICF cell subsets, their marker genes, and special functions compared to other groups. By analyzing marker genes from special ICF subgroups, we identified prognosis-related genes. Finally, Lasso modelling was used to create a risk model with 10 signature genes that can be used to figure out the prognosis of COAD. These genes included ANGPTL-4, TIMP1, SNAI1, S1PR5, ADAM9, CMC1, ETS2, ALDH2, SER PINA1, and ASAH1. The model was consistent in validating the TCGA training set, the entire set, and two GEO external validation sets. The model’s score was confirmed to be an independent prognostic factor using univariate and multivariate Cox analyses.

Angiopoietin-like Protein 4 (ANGPTL4) is a new adipocyte factor that controls the metabolism of fats and impacts the body's energy balance [48, 49]. ANGPTL-4 is involved in vascular permeability, angiogenesis, and inflammatory signal transduction and may also play a role in tumour progression [50-52]. TME can be altered by inflammatory adipose tissue, which may explain the relationship with several tumours [53-56]. ANGPTL4 has been linked to the development of human colorectal cancer, particularly venous invasion and distant metastasis [57]. Through ANGPTL4, prostaglandin E2 helps COAD cells adapt to low oxygen levels and the growth of cancer [58]. Tissue Inhibitor of Metalloproteinase 1 (TIMP1) is a metalloproteinase from the tissue inhibitor family. TIMP1 overexpression is linked to a poor prognosis in several tumours [59-62]. Several studies have shown that high levels of TIMP1 are found in colon cancer tissues and lymph node metastases compared to normal tissues [63]. The results have made it clear that blocking TIMP1 expression stops cells from growing and helps them die by turning on the FAK-PI3K/AKT and MAPK signalling pathways, which are mainly controlled by TIMP1 [64]. TIMP1 likely has a significant function in facilitating the development of tumours in humans and the spread of COAD to other parts of the body. It has the potential to serve as a prognostic biomarker for COAD [65]. TIMP1 may also play a role in COAD chemotherapeutic resistance, making it a potential target for overcoming 5-fluorouracil resistance [66]. Snail family translational reporter 1 (SNAI1) is a transcription factor that controls the change from epithelial to mesenchymal in COAD tissue, which starts the invasion of tumour cells [67]. CRC cells become radiation resistant when SNAI1 is overexpressed [68]. Sphingosine 1-phosphate Receptor 5 (S1PR5) is a component of the chemokine receptor in NK cells. It regulates the *in vivo* distribution of NK lymphocytes and lesion site transport [69]. It is expressed most prominently in cells with immune function [70]. It is strongly linked to immune cell transport, cell survival, cell proliferation, cell migration, angiogenesis, and cancer progression [71, 72]. COAD promotes tumour growth, migration, and invasion by activating the NF-κB/IDO1 signal pathway [73]. We have observed more ADAM9 mRNA in COAD tissue, and that ADAM9 overexpression raises the level of epithelial cadherin in HT29 cells from a human recovered COAD. This happens through growth factors. The secreted form of ADAM9 aids in the invasion and progression of COAD cells through tumor matrix interaction [74-76]. ETS-2 is a member of the transcription factor family [77]. The increased expression of ETS protein is related to the disease progression of many tumours, including CRC [78, 79]. It has been identified as a Wnt target of CRC cells and intestinal stem cells, and it can regulate colon stem cell sensitivity and tumour growth [80, 81]. Acetaldehyde produced during alcohol metabolism is a recognized animal carcinogen, most of which is eliminated by liver mitochondrial Aldehyde Dehydrogenase 2 (ALDH2). More than 40% of Japanese people have inactive ALDH2, increasing their risk of esophageal and head and neck cancers [82]. Alcohol consumption has been linked to CRC in epidemiological studies. The ALDH2* *gene polymorphism may influence the risk of CRC. Different ALDH2 genotypes have different preventive effects on reducing alcohol concentration [83]. Therefore, most of the 10 genes constituting this marker are related to the pathogenesis and progression of COAD and can be considered potential therapeutic targets. However, there are only a few genes related to immunity. The relationship between CMC1*,* SERPINA1, and ASAH1 genes and tumours has not been studied; this is the next step we should take. This work focused on analysing the expression profiles of model genes that have not been previously published. Our findings have revealed the expression levels of CMC1, SERPINA1, and ASAH1. The results of the functional experiment have shown that decreasing the expression of S1PR5 made it harder for SW620 cells to divide in the lab, but increasing the expression of CMC1 and ASAH1 made it easier for SW480 cells to divide in the lab. The data demonstrated that the newly developed prognostic model has the potential to be applied in clinical settings and could identify possible therapeutic targets for patients with CRC.

TME and immune cells play a critical role in tumor prognosis. The main co-enrichment pathways revealed by enrichment analysis were interferon response, allograft rejection, TNFA signal, and others. Simultaneously, significant differences in multiple indicators have been found to exist between the high-risk groups, indicating the model to be significantly related to multiple cancer characteristics. Finally, we found differences in immune responses between high-risk groups using drug sensitivity analysis and immunotherapy response analysis. The model score, which may be the marker of the immune response, could predict the benefits of immunotherapy. In addition, the low-risk group exhibited two distinct types of IPS scores that were considerably higher than those of the high-risk group. This suggests that patients in the low-risk group were more prone to experiencing positive outcomes following immunotherapy. Based on the findings, the group with a lower risk was more inclined to derive advantages from immunotherapy. Thus, the model could serve as a dependable prognostic tool for COAD immunotherapy.

This study holds significant potential for clinical application as it can assess prognostic risk, identify potential treatment targets, and guide personalised therapeutic strategies for patients with COAD. It would be critical to address the knowledge gaps and further validate these findings through prospective studies and comprehensive functional analyses. Translating these models from research to clinical practice would require extensive validation across diverse patient populations and settings. The differences in sequencing platforms, sample preparation methods, and analytical pipelines can introduce variability, further complicating the standardization of these models. Moreover, retrospective studies, which form the basis for many current models, may not fully capture the dynamics of real-world clinical settings, necessitating prospective studies for validation. Looking forward, this area is poised to evolve significantly in the next five years, driven by advancements in molecular profiling and immunotherapy, potentially leading to more personalised and effective CRC management.

We recognise some limitations of this study. First, because our data were derived from a retrospective dataset, there may be an inherent risk of bias, and the findings may not fully represent the broader patient population. To address this, future studies should incorporate prospective research to validate the effectiveness and generalisability of the results. Additionally, the value of gene expression might be affected by sampling bias due to variations across different platforms, emphasising the need for standardised methodologies. Second, we could not be sure of the link between the gene signature found and immunotherapy reactivity because we did not have any data from patients who were treated with immune checkpoint inhibitors. Further research, including clinical trials involving these therapies, is necessary to confirm the model's applicability in this context. Finally, this study has primarily focused on microarray tabular datasets, which, while informative, do not capture the full complexity of COAD. More in-depth cell experiments, animal studies, and comprehensive analyses incorporating multi-omics data should be conducted to validate the model’s prognostic and therapeutic potential in COAD patients. Adding important clinical and pathological factors related to COAD, like levels of carcinoembryonic antigen, MSI, dMMR status, and gene mutations in KRAS, NRAS, and BRAF, could prove to be very important in future studies to improve the model's ability to predict accurately and be useful in clinical settings.

## CONCLUSION

We have, herein, developed a risk model utilising scRNA-seq and bulk RNA-seq data to forecast the prognosis and effectiveness of immunotherapy for COAD patients. This study has identified four synergistic prognostic ICF gene sets in the TCGA-COAD cohort. The single-cell dataset has been analyzed to identify prognosis-related ICF cell subsets, their marker genes, and special functions compared to other groups. By analyzing marker genes from special ICF subgroups, we have identified prognosis-related genes. Finally, Lasso modelling has been used to create a risk model with 10 signature genes that can be used to figure out the prognosis of COAD. These genes have included ANGPTL-4, TIMP1, SNAI1, S1PR5, ADAM9, CMC1, ETS2, ALDH2, SER PINA1, and ASAH1. The model has been found to be consistent in validating the TCGA training set, the entire set, and two GEO external validation sets. The model’s score has been confirmed to be an independent prognostic factor using univariate and multivariate Cox analyses. In addition, we have discovered three model genes (S1PR5, CMC1, and ASAH1) that have not been reported before. qPCR and immunohistochemistry analyses have revealed S1PR5 expression to be high in COAD tissues, while CMC1 and ASAH1 expressions were low. According to the findings of the functional experiment, S1PR5, CMC1, and ASAH1 may control the ability of CRC cells to proliferate. These genes have been found to possess the potential to serve as new therapeutic targets in CRC. These findings indicate that this model has the ability to assess the prognostic risk and identify possible treatment targets for patients with COAD.

## Figures and Tables

**Fig. (1):**
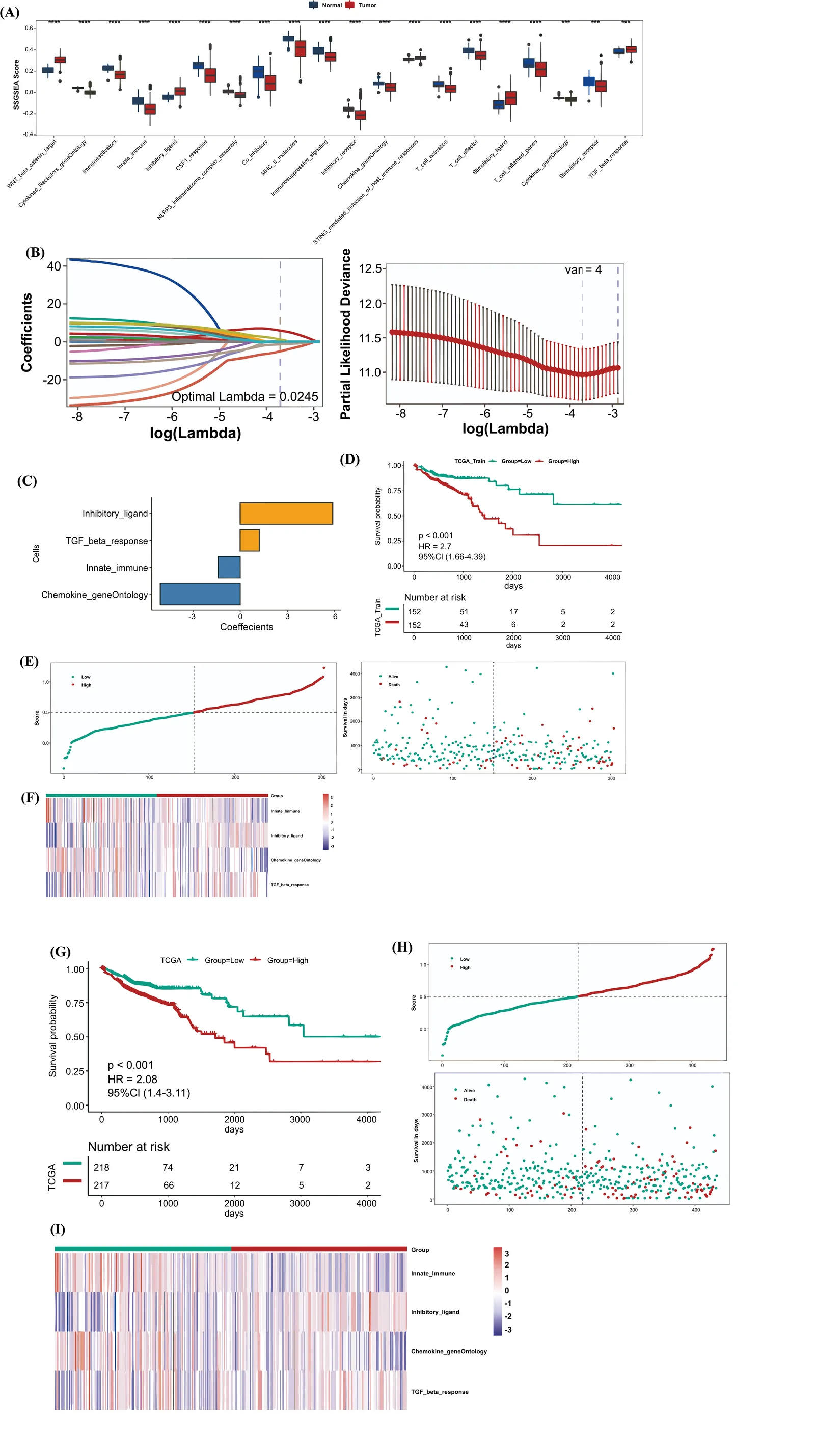
Construction and evaluation of TCGA dataset ICFRS. **A**) Enrichment score box chart of differentially enriched ICF in tumor_*versus*_normal groups. **B**) Change track of the independent variable in Lasso regression. **C**) The numbers on the left show the logarithm of the independent variable lambda and the numbers on the right show the coefficient of the independent variable and the confidence interval under each lambda of Lasso regression. **D**) The numbers on the right show the critical prognosis ICF and the curves for survival between high- and low-ICFRS groups in the TCGA training set. **E**) The numbers on the left show the, risk triple diagram of the TCGA training set, which includes the risk dispersion point diagram, the survival time scatter point diagram, and the ICF enrichment score heatmap in the risk score grouping. **F**) Red colour represents the high-risk group, and green colour represents the low-risk group. **G**) Survival curve between high- and low-ICFRS groups in the TCGA cohort. **H**, **I**) The risk triple diagram of the TCGA’s overall set, which is, respectively, the risk dispersion point diagram, the survival time scatter point diagram, and the ICF enrichment score heatmap in the risk score grouping. The red colour represents the high-risk group, and the green colour represents the low-risk group.

**Fig. (2):**
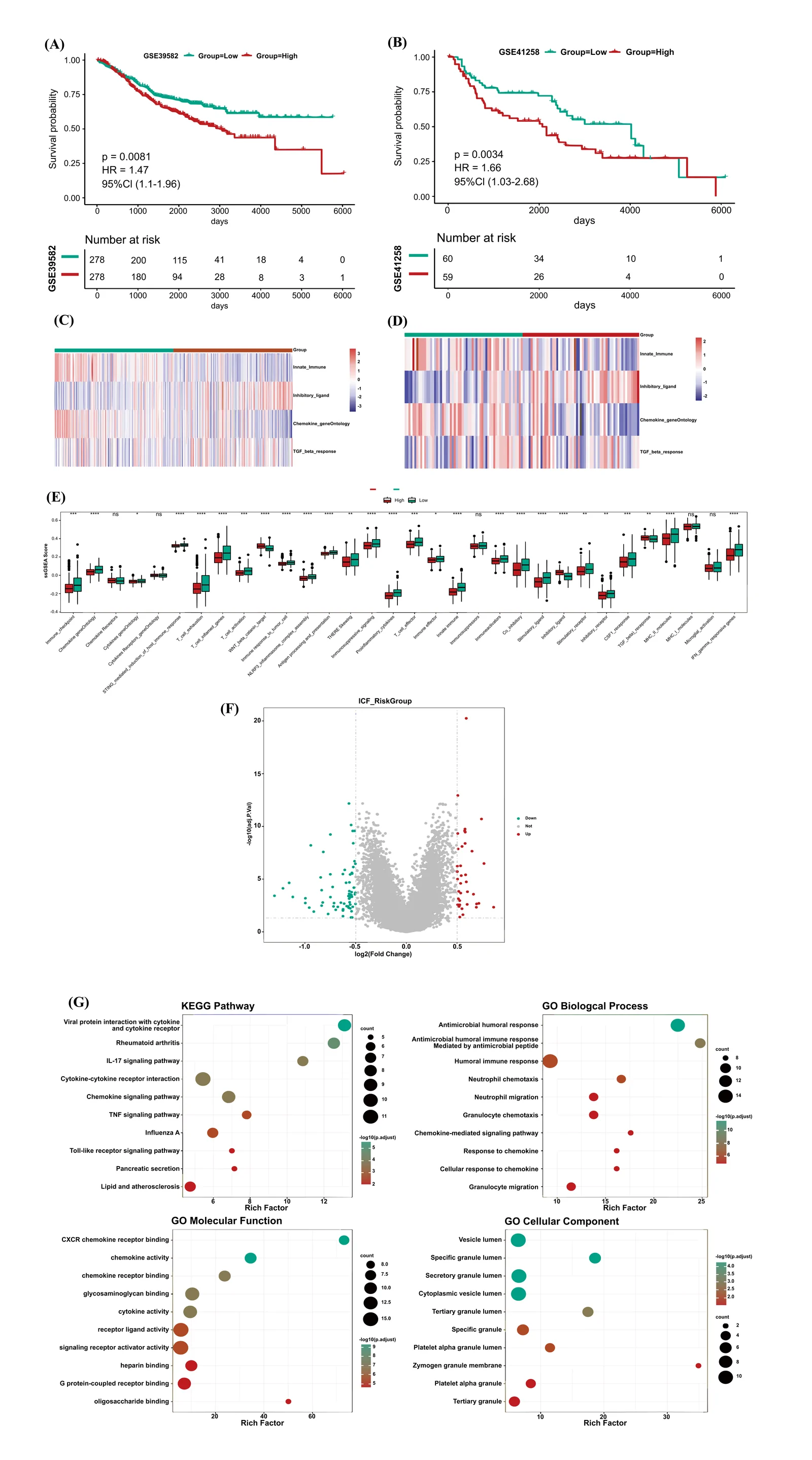
Verification and evaluation of the TCGA ICFRS. (**A**, **B**) Kaplan-Meier curves of two GEO external validation sets; red colour represents the high-risk group and green colour represents the low-risk group. (**C**, **D**) This is a heatmap that shows the difference in the ICF enrichment score between high-risk and low-risk groups after centralised validation with two GEO validation sets. **E**) The enrichment difference of 33 immune function gene sets in the TCGA dataset between high- and low-ICFRS groups. (**F**, **G**) This is a volcano map of DEGs; red colour means up-regulation, green means down-regulation, and grey means there is not a significant difference in expression; this is a bubble diagram of KEGG pathway enrichment and GO function enrichment of DEGs between the high-risk and low-risk groups. The colour of the dots shows how significant the enrichment is, and the size of the dots shows how many DEGs are enriched.

**Fig. (3):**
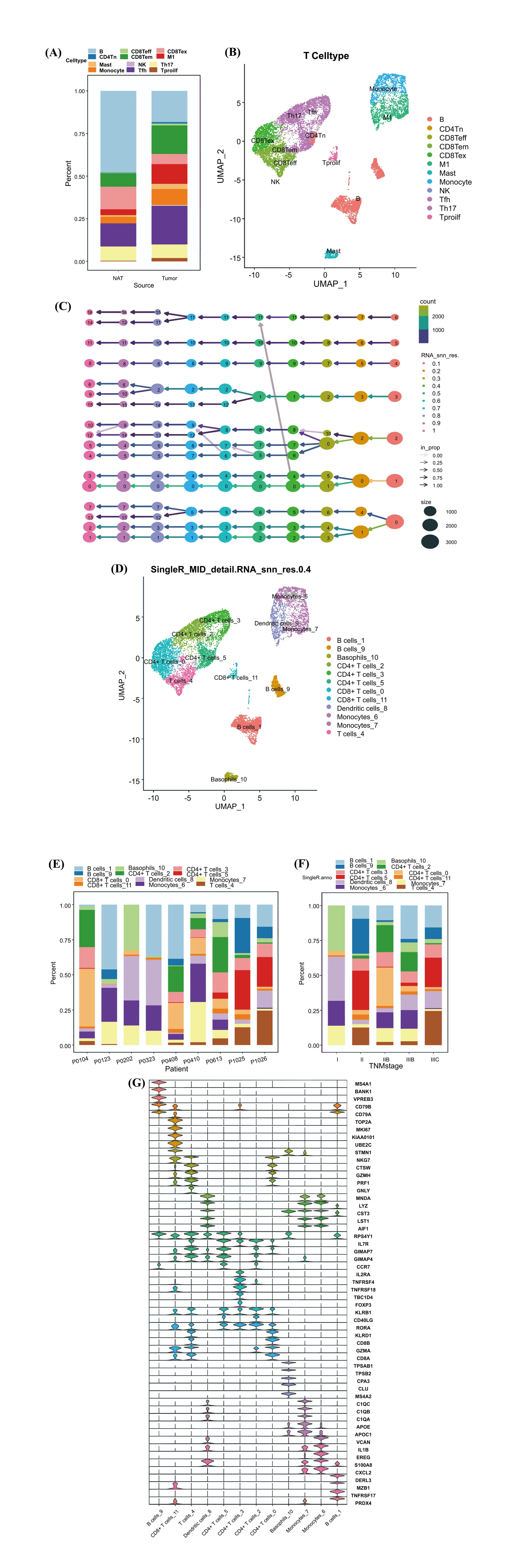
Recognition of tumour single-cell subsets. **A**) A histogram of the difference in the cumulative distribution of cells between normal and tumour tissues. **B**) UMAP map of cell groups annotated in the original dataset. (**C**, **D**) SingleR-annotated UMAP diagram of cell subgroup distribution. **E**) Cumulative distribution histogram of cell types among different patients. **F**) Cumulative distribution histogram of cell types among patients with different TNM stages. **G**) Violin map of top 5 marker genes expression of the identified cell subgroup in this analysis.

**Fig. (5):**
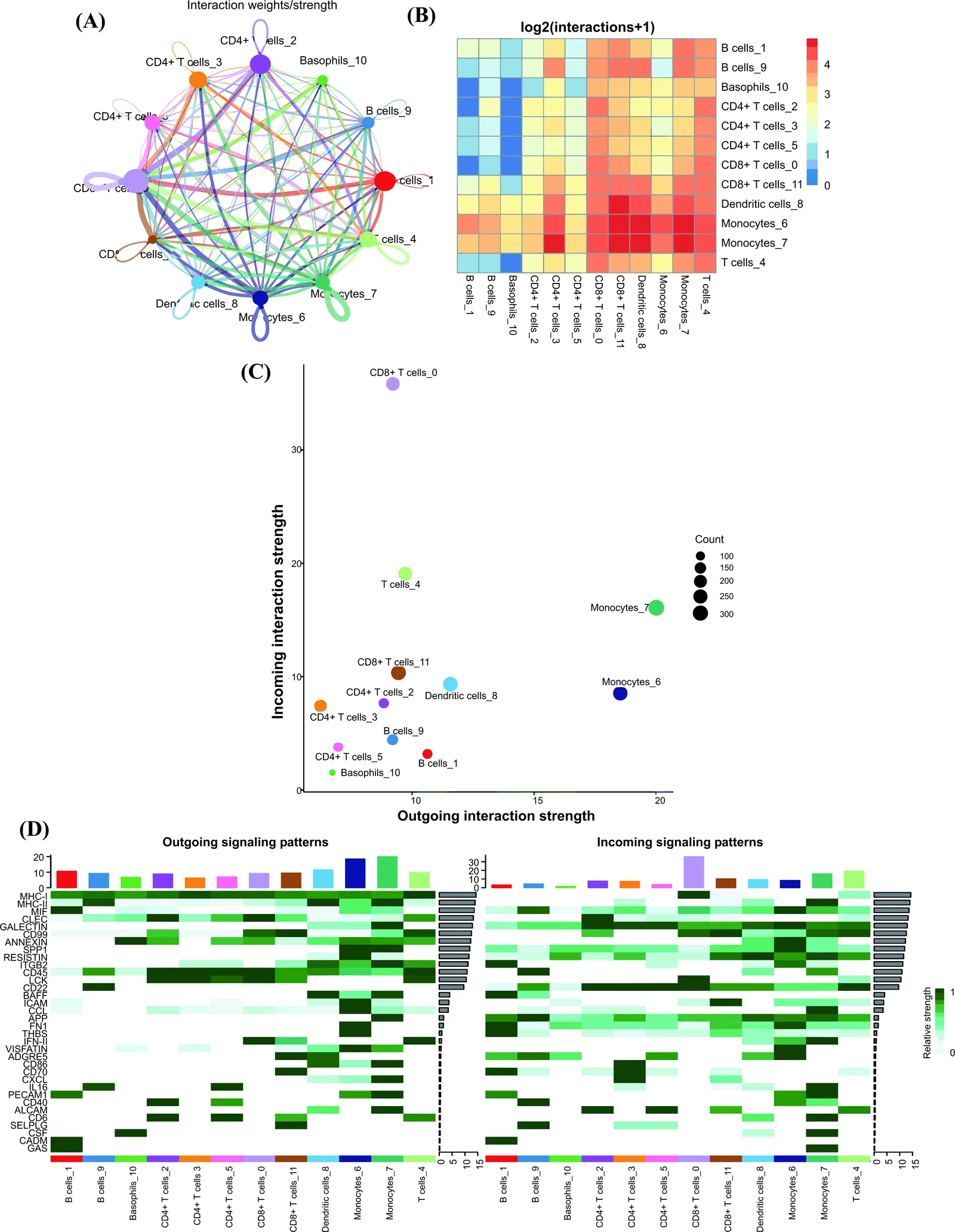
Cell communication among cell subsets. **A**) Cell communication network diagram. The solid circles with different colours represent different cell groups. (**B**, **C**) The solid circle's size is proportional to the number of cells in the cell group. Each side's color corresponds to the signal sender, while the thickness of the side reflects the intensity of communication. The x-axis and y-axis indicate the strength of the cell group as a signal sender and receiver, respectively. The dominant signal heatmap. **D**) identifies the global communication mode and main signals of each cell group.

**Fig. (6):**
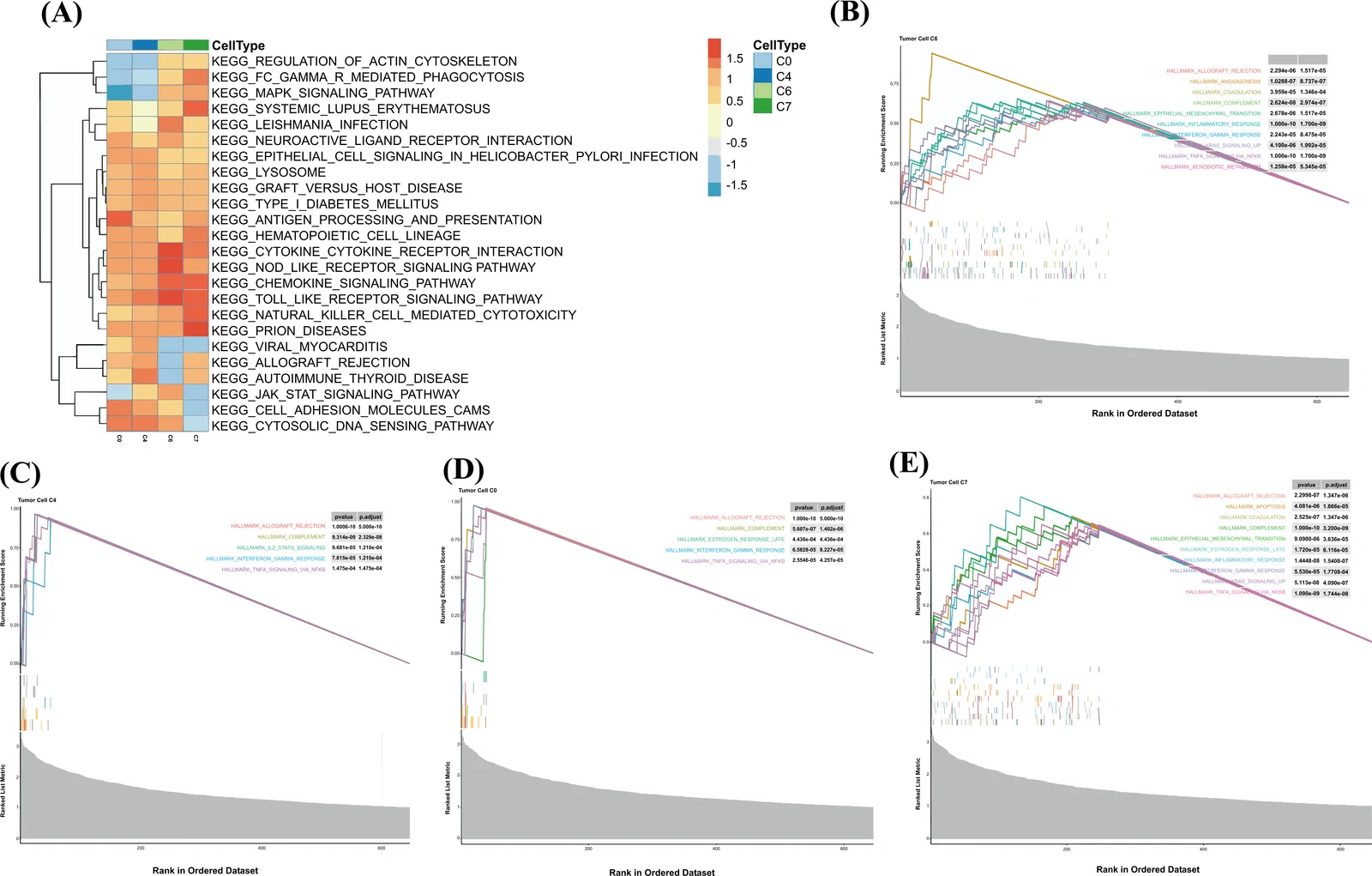
Functional identification of a 4-immune ICF cell population. **A**) A heatmap of the KEGG pathway enrichment NES value of the ICF cell subgroup marker gene in the prognosis. **B**-**E**) A line graph of the GSEA enrichment score of the HALLMARK pathway of four subgroups.

**Fig. (7):**
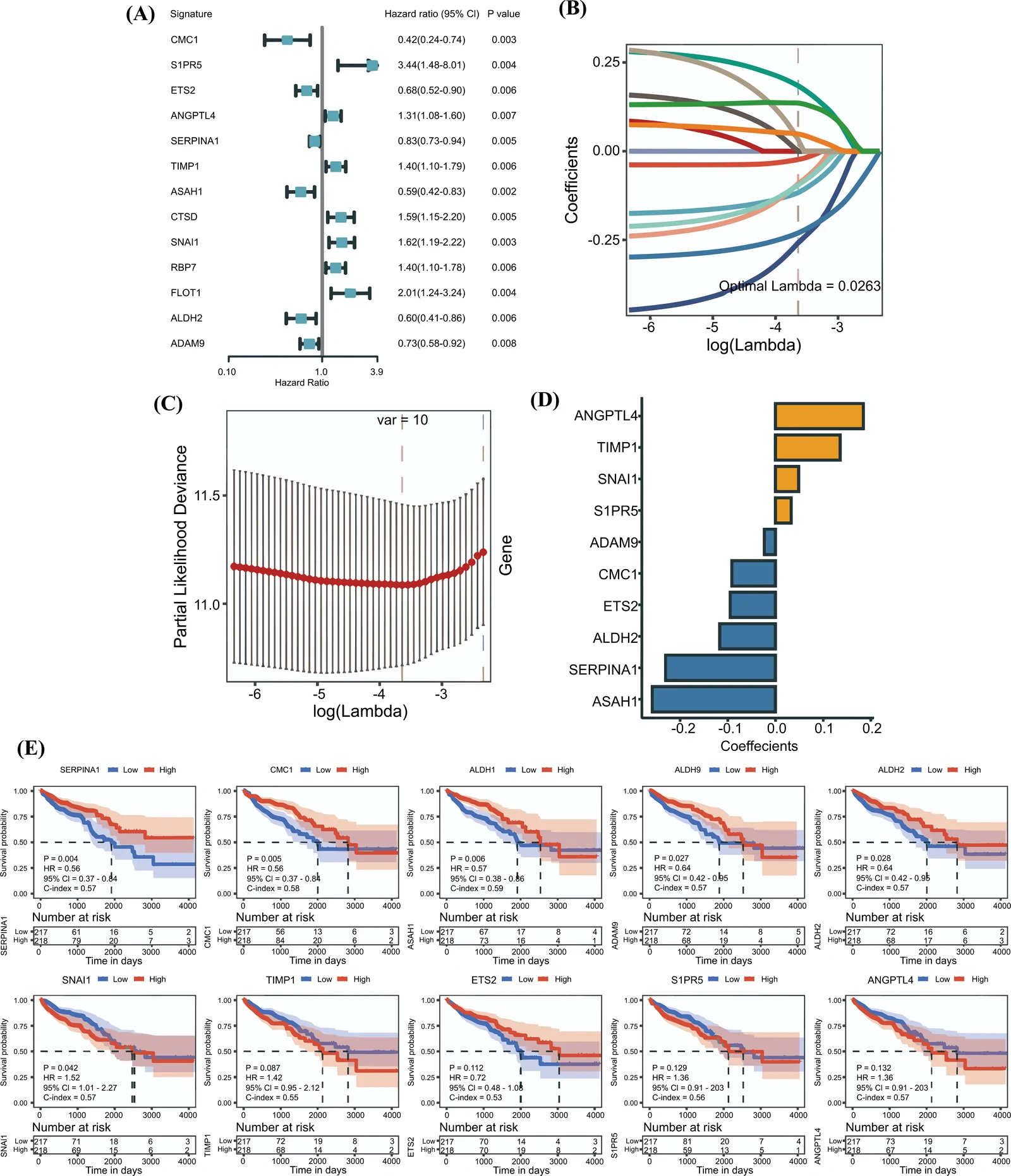
Lasso regression analysis of The Cancer Genome Atlas dataset has yielded the following results. **A**) The forest map shows the results of a one-variable Cox analysis. **B**) The ordinate shows the coefficient of the independent variable and the abscissa shows the logarithm of the independent variable lambda. **C**) The confidence interval for Lasso regression under each lambda is shown. **D**) The Lasso regression coefficient of key prognostic genes is shown. **E**) A Kaplan-Meier curve of the prognosis signature obtained using Lasso analysis, with red representing the high expression group and blue representing the low expression group.

**Fig. (8):**
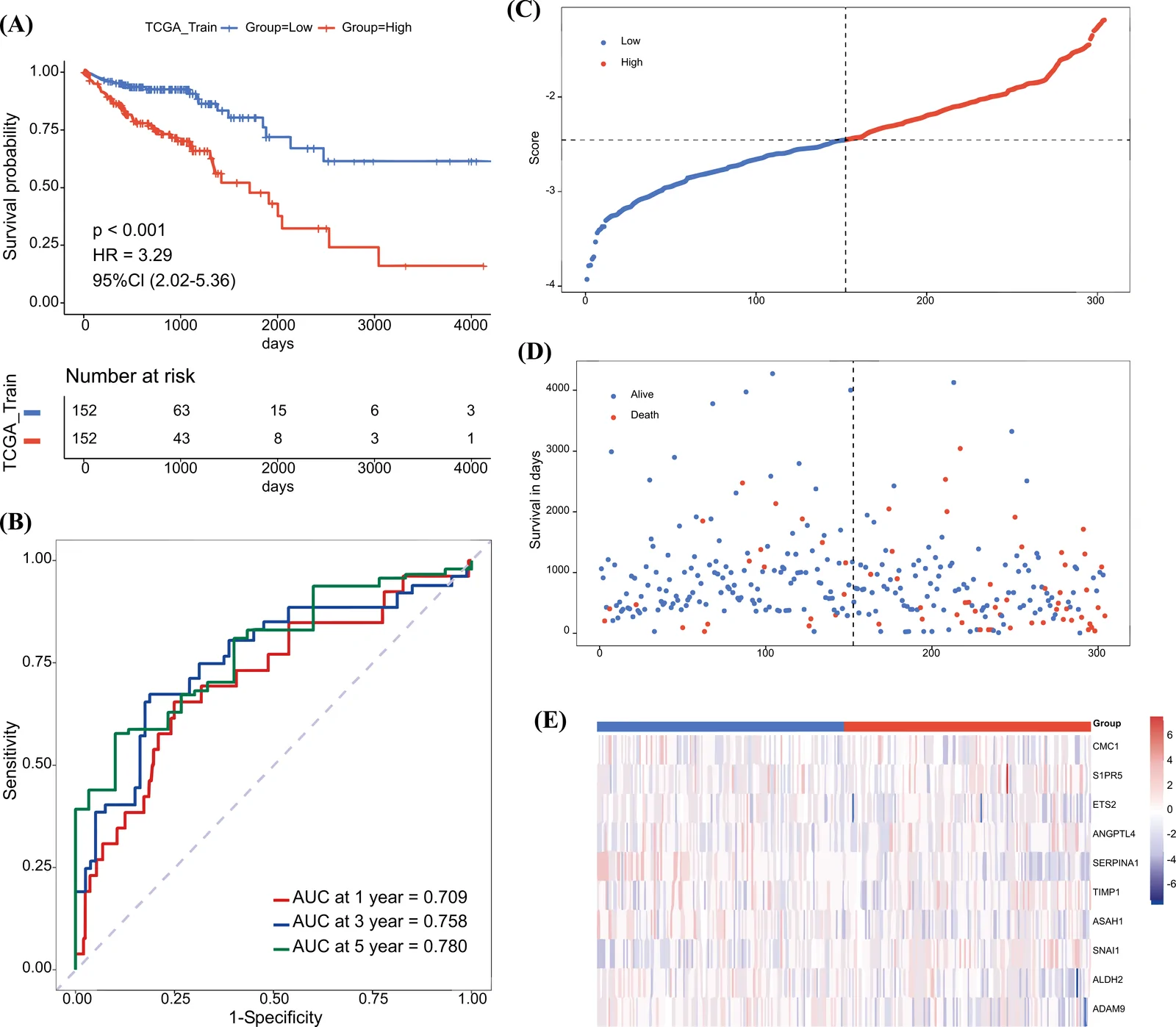
The prognostic efficacy of the TCGA training set validation model. The Cancer Genome Atlas training set illustrating a Kaplan-Meier curve. **A**), a receiver operating characteristic curve (**B**), and a risk triple diagram (**C**-**E**). The risk triple diagram includes a risk dispersion point diagram, a survival time dispersion point diagram, and an expression heat diagram of model genes in the RS grouping. The red colour represents the high-risk group, while the blue colour represents the low-risk group.

**Fig. (11):**
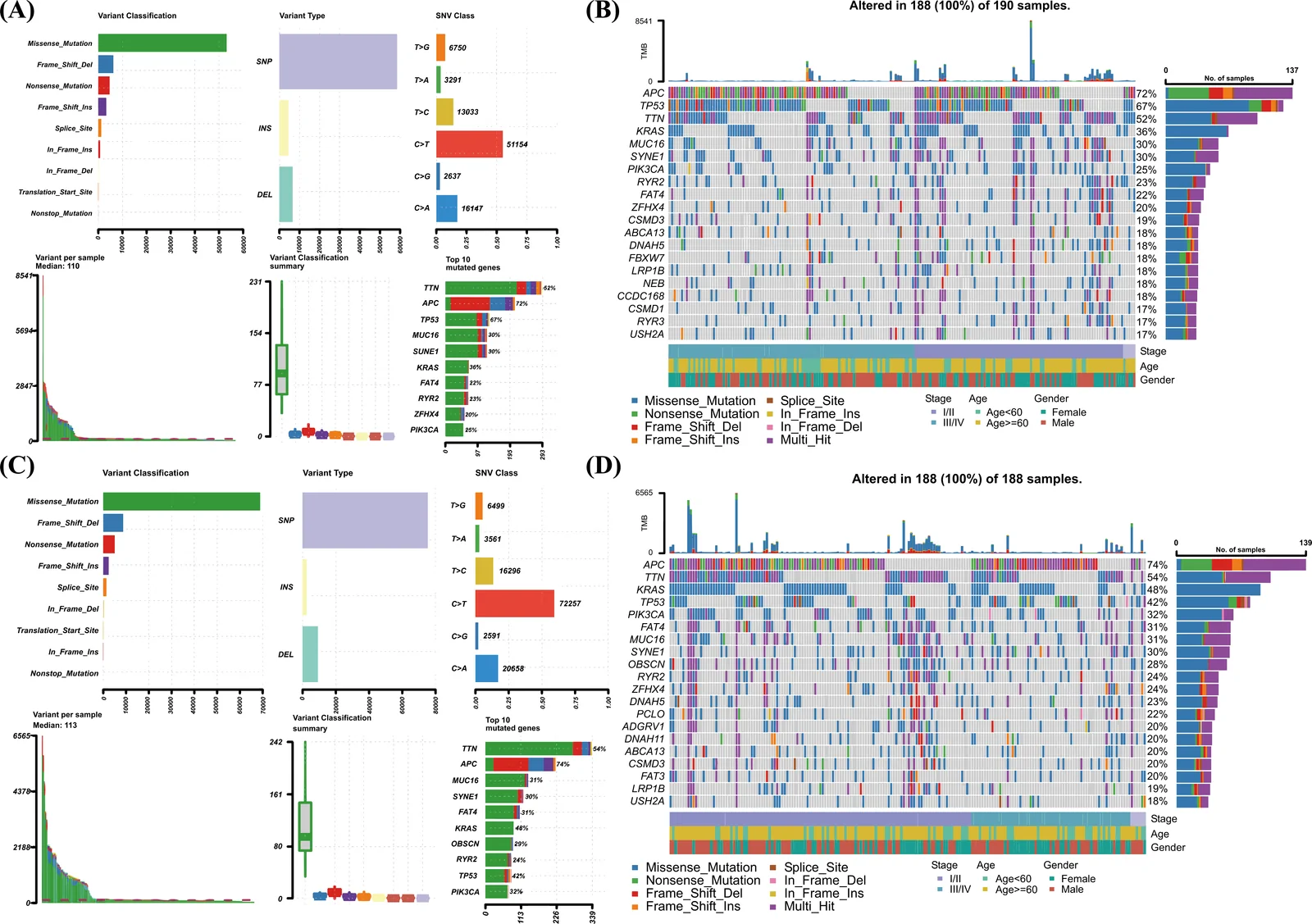
(**A**-**C**): Mutation summary chart for high- and low-risk groups. (**B**) SNV waterfall diagram of top 20 (mutation frequency) genes in the high-risk group. (**D**) SNV waterfall of top 20 (mutation frequency) genes in the low-risk group.

**Fig. (12):**
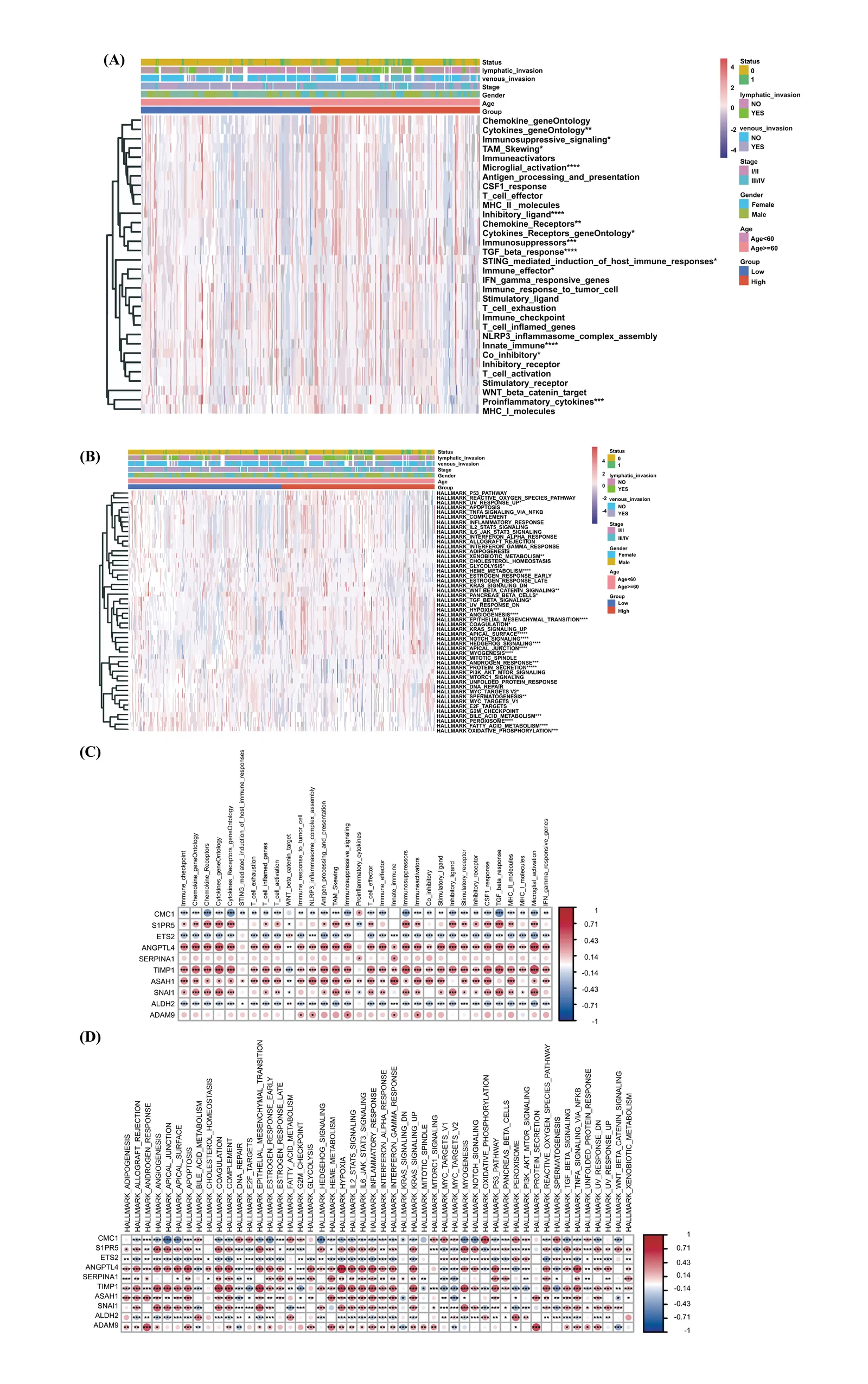
Differences in pathway enrichment between model groups. The difference between the immune function gene set and the HALLMARK pathway enrichment score in the high-risk and low-risk groups is shown in (**A**, **B**). The asterisk (*) indicates how important the difference is. (**C**, **D**) A heatmap showing the relationship among model gene expression, the immune function gene set, and the KEGG pathway enrichment score. The colour depth shows the strength of the relationship, and an asterisk (*) means it is important.

**Fig. (13):**
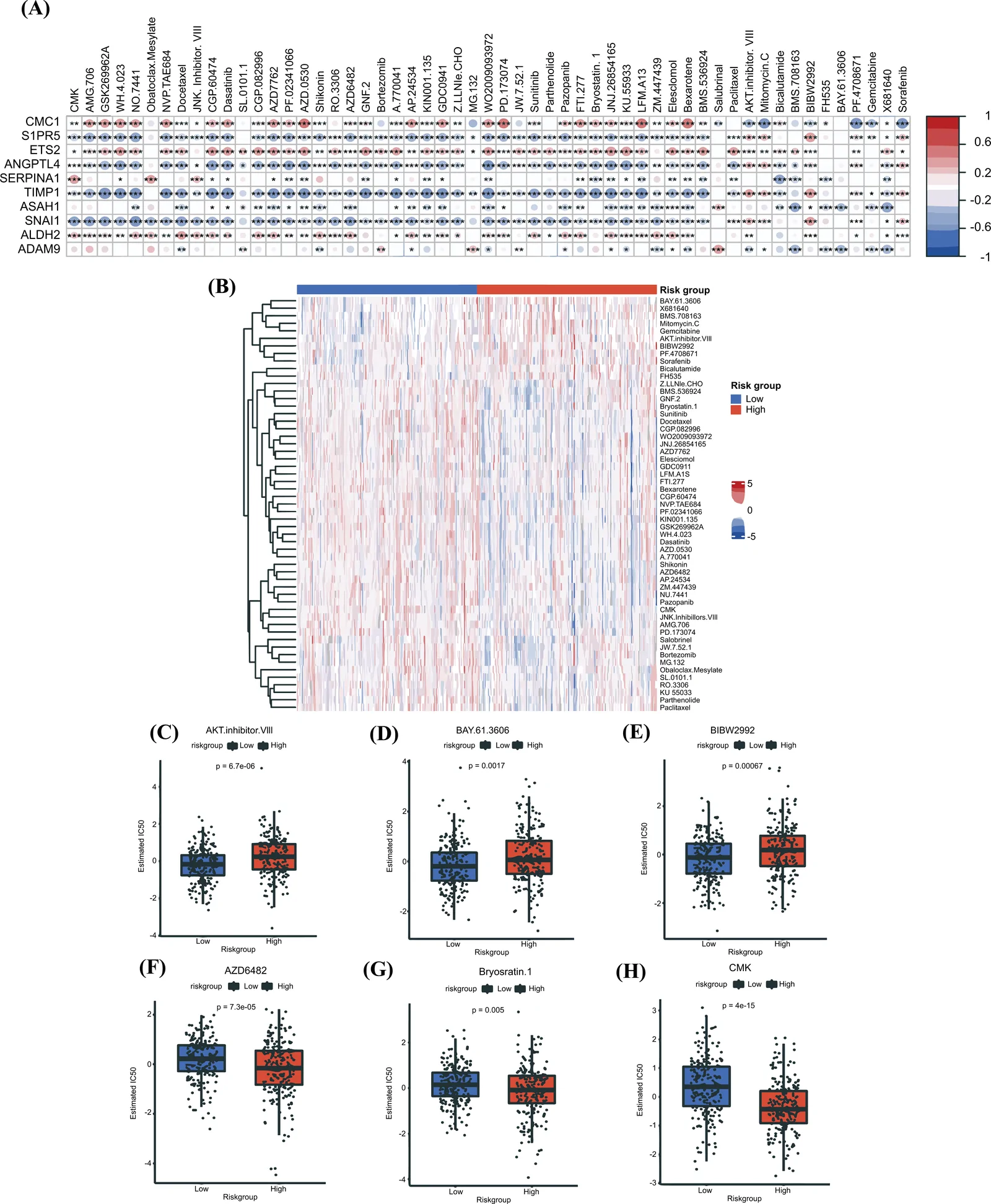
The RS Model for Predicting Chemotherapeutic Drug Resistance. (**A**) A heatmap of the correlation between model gene expression and drug IC_50_ value. The dots’ colour depth represents the correlation level, and the * represents significance. (**B**) The IC_50_ values of sensitive and different drugs are assigned to high- and low-risk groups. (**C**-**H**) The distribution of IC_50_ values for chemotherapy drugs differs between high- and low-risk groups, with red denoting the high-risk group and blue the low-risk group.

**Fig. (14):**
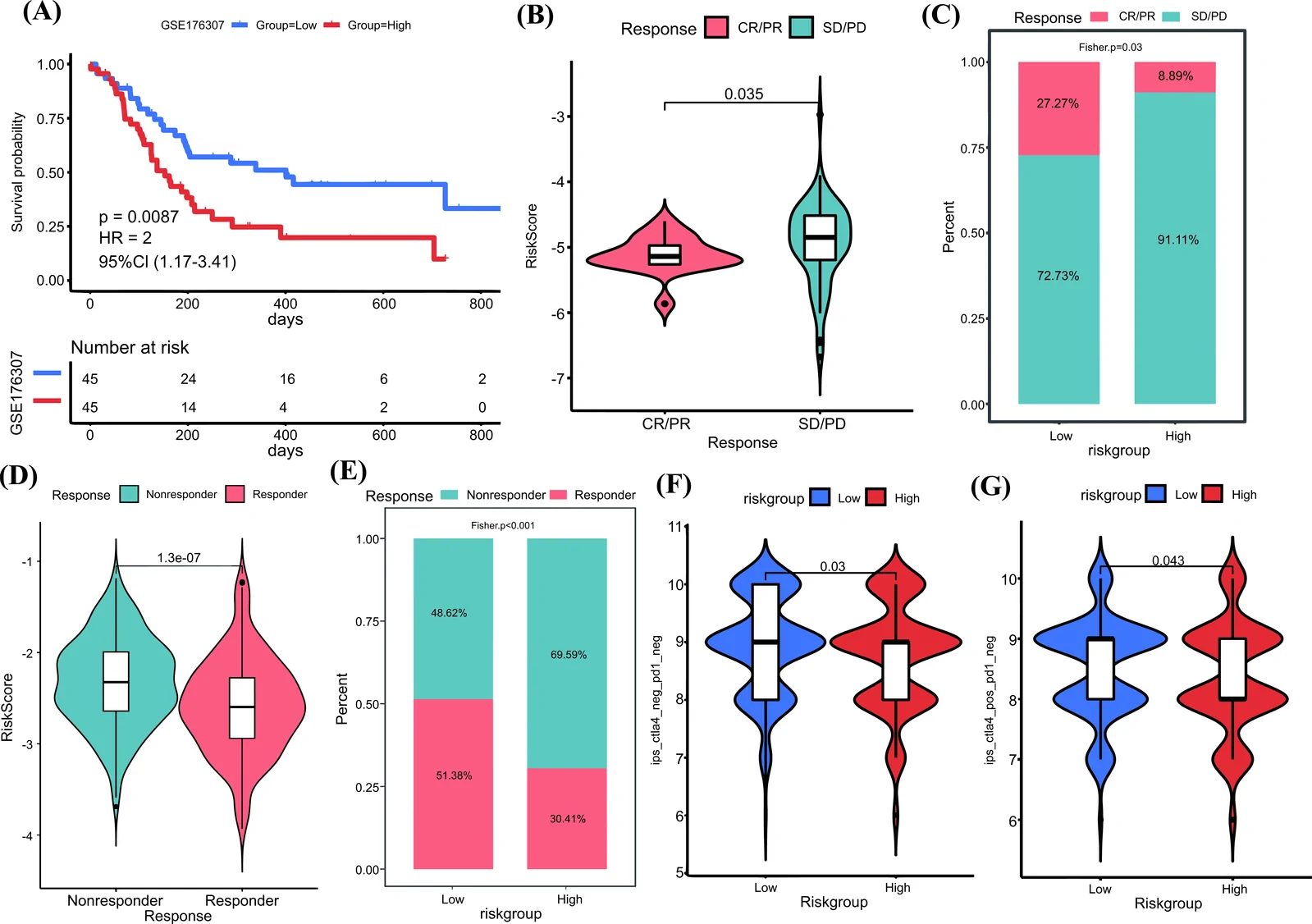
RS model for predicting therapeutic effects on the patient. **A**) The Kaplan-Meier curve of the high- and low-risk groups in the immunotherapy cohort. **B**) The RS distribution difference violin chart of the immunotherapy cohort with or without response group. **C**) The histogram illustrates the cumulative distribution difference between high-risk groups in the immunotherapy cohort, both with and without a response. **D**) The RS distribution violin diagram illustrates the varying immunotherapy response groups in the TCGA cohort, as predicted by TIDE. **E**) The cumulative partial histogram illustrates the immune response of the TCGA cohort, with red colour denoting a response and blue colour denoting no response. (**F**, **G**) IPS score difference between the high- and low-risk groups of the TCGA queue; red colour represents the high-risk group, and blue colour represents the low-risk group.

## Data Availability

All data generated or analysed during this study are included in this published article.

## LIST OF ABBREVIATIONS

COAD: Colon Adenocarcinoma
ICF: Immune Cell Functions
CRC: Colorectal Cancer
TME: Tumour Microenvironment
TCGA: The Cancer Genome Atlas
DEGs: Differentially Expressed Genes
RS: Risk Score
GEO: Gene Expression Omnibus
