# Supplementary material for: The Risk Genes S1PR5, CMC1, and ASAH1 as Potential Targets for the Diagnosis, Immunotherapy, and Treatment of Colon Adenocarcinoma by Single-Cell and Bulk RNA Sequencing Analysis
Source: Curr Med Chem. 2024 Nov 7;33(16):3211–45. doi: 10.2174/0109298673331144241009082052 (PMC13555847; doi:10.2174/0109298673331144241009082052)
Supplement: Supplementary file 1 [file CMC-33-16-3211_SD1.pdf]

Supplementary Material

The Risk Genes SIRP5, CMC1, and ASAH1 as Potential Targets for the Diagnosis, Immunotherapy, and Treatment of Colon Adenocarcinoma by Single-Cell and Bulk RNA Sequencing Analysis

Zipeng Xu[1,#], Jiantao Gong[2,#], Weidong Hu[1], Chen Ge[1], Genxi Tong[1], Fengjun Cai[1], Zhenghai Zhu[1], Yihang Yuan[3,*] and Chaobo Chen[1,*]

[1]Department of General Surgery, Xishan People's Hospital of Wuxi City, Wuxi, 214105, China; [2]Department of Gastroenterology, Xishan People's Hospital of Wuxi City, Wuxi, 214105, China; [3]Department of General Surgery, Nanjing Drum Tower Hospital, Affiliated Hospital of Medical School, Nanjing University, Nanjing, 210008 China

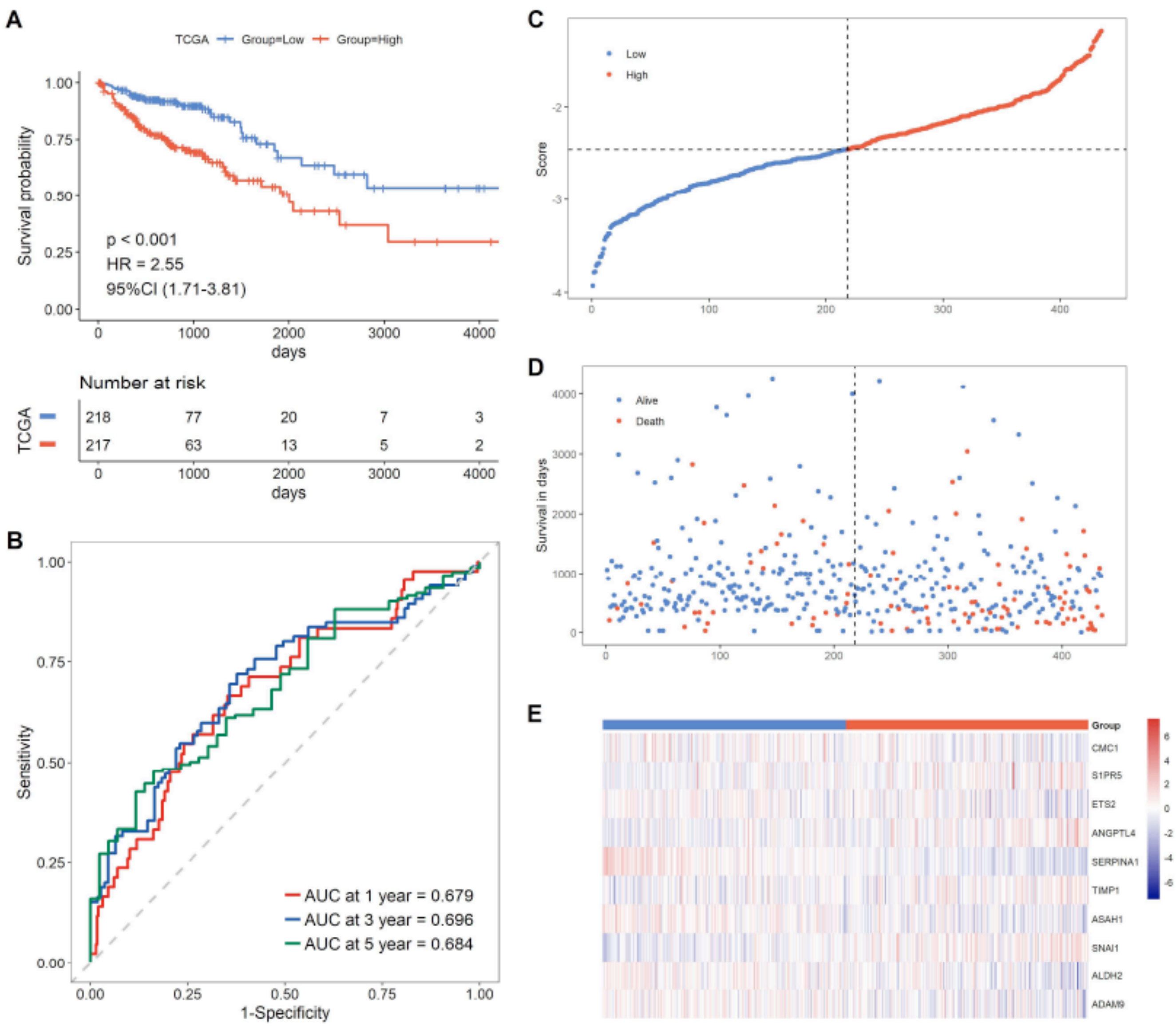

Supplementary Fig. (1). Results of TCGA ensemble validation model effectiveness. A-B: The Kaplan-Meier curve and receiver operating characteristic curve of the TCGA ensemble. C-E: The risk triplet of the TCGA ensemble, including the risk gain scatter plot, the survival time scatter plot, and the expression heat plot of model genes in the RS grouping.

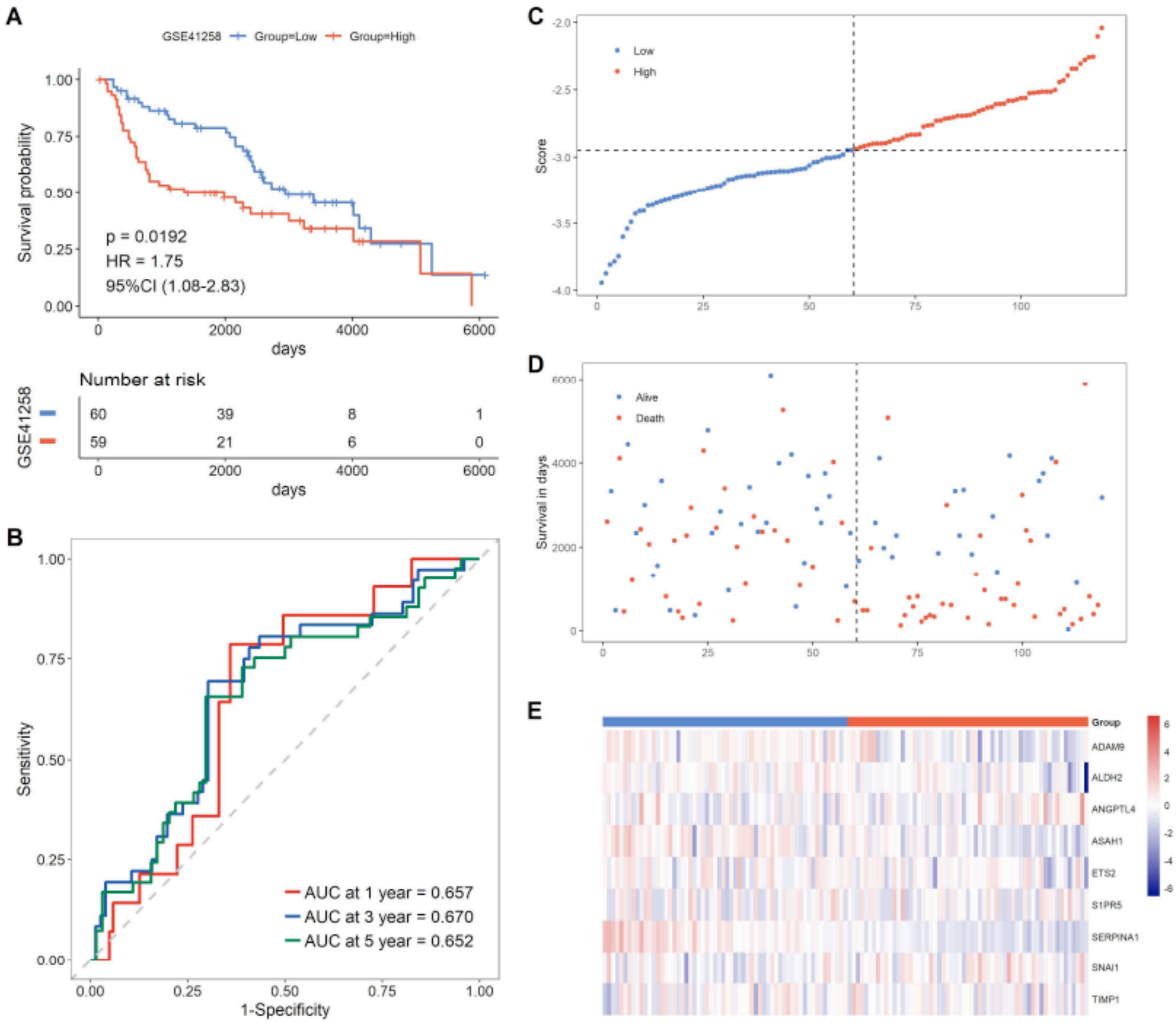

Supplementary Fig. (2). Results of GSE41258 validation set. The GSE41258 dataset included a Kaplan-Meier curve and an ROC curve (A–B). The risk triple diagram (C–E) shows the risk dispersion point diagram, the survival time dispersion point diagram, and the expression heat diagram of model genes in the risk score grouping. The red colour represents the high-risk group, while the blue colour represents the low-risk group.

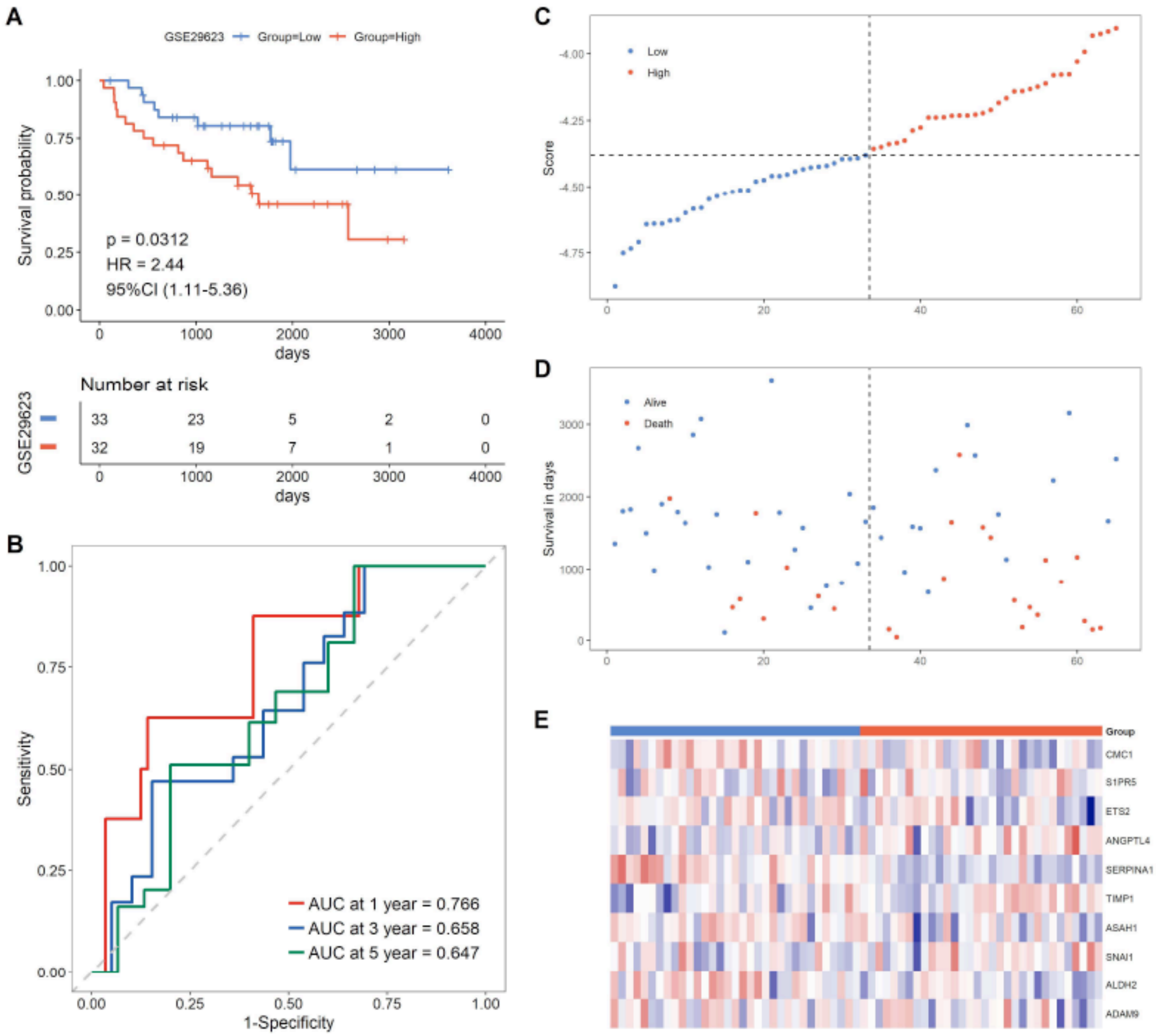

Supplementary Fig. (3). Results of GSE29623 validation set. The GSE29623 dataset includes the Kaplan-Meier curve and ROC curve. The risk triple diagram includes the risk dispersion point diagram, the survival time dispersion point diagram, and the expression heat diagram of model genes in the RS grouping. Each of these is shown in A–E. The red colour represents the high-risk group, while the blue colour represents the low-risk group.
